# Supplementary material for: Surface passivation for highly active, selective, stable, and scalable CO2 electroreduction
Source: Nat Commun. 2023 Aug 3;14:4670. doi: 10.1038/s41467-023-40342-6 (PMC10400642; doi:10.1038/s41467-023-40342-6)
Supplement: Supplementary file 1 — Supplementary Information [file 41467_2023_40342_MOESM1_ESM.pdf]

Supplementary Information:

Surface passivation for highly active, selective, stable, and scalable CO_2 electroreduction

Jiexin Zhu[1,2,†], Jiantao Li[1,†], Ruihu Lu[3,†], Ruohan Yu[1,†], Shiyong Zhai[4], Chengbo Li[5], Lei Lv[1], Lixue Xia[6], Xingbao Chen[1], Wenwei Cai[1], Jiashen Meng[1,7], Wei Zhang[1], Xuelei Pan[1], Xufeng Hong[7], Yuhang Dai[1,2], Yu Mao[3], Jiong Li[8], Liang Zhou[1,9], Guanjie He[8], Quanquan Pang[7], Yan Zhao[6], Chuan Xia[5,*], Ziyun Wang[3,*], Liming Dai[4,*], Liqiang Mai[1,9,*]

[1]State Key Laboratory of Advanced Technology for Materials Synthesis and Processing, Wuhan University of Technology, Wuhan 430070, Hubei, P. R. China.

[2]Electrochemical Innovation Lab, Department of Chemical Engineering, University College London, London, WC1E 7JE, UK

[3]School of Chemical Sciences, The University of Auckland, Auckland, 1010, New Zealand.

[4]Australian Carbon Materials Centre (A-CMC), School of Chemical Engineering, University of New South Wales, Sydney, NSW, 2052, Australia

[5]School of Materials and Energy, University of Electronic Science and Technology of China, Chengdu, P. R. China.

[6]International School of Materials Science and Engineering, Wuhan University of Technology, Wuhan 430070, Hubei, P. R. China.

[7]Beijing Key Laboratory for Theory and Technology of Advanced Battery Materials, School of Materials Science and Engineering, Peking University, Beijing 100871, P. R. China.

[8]Shanghai Synchrotron Radiation Facility, Shanghai Advanced Research Institute, Chinese Academy of Sciences, Shanghai, 201210 P. R. China

[9]Hubei Longzhong Laboratory, Wuhan University of Technology (Xiangyang Demonstration Zone), Xiangyang 441000, Hubei, P. R. China.

[†]This authors contributed equally to this work.

*Email: chuan.xia@uestc.edu.cn; ziyun.wang@auckland.ac.nz; l.dai@unsw.edu.au; mlq518@whut.edu.cn

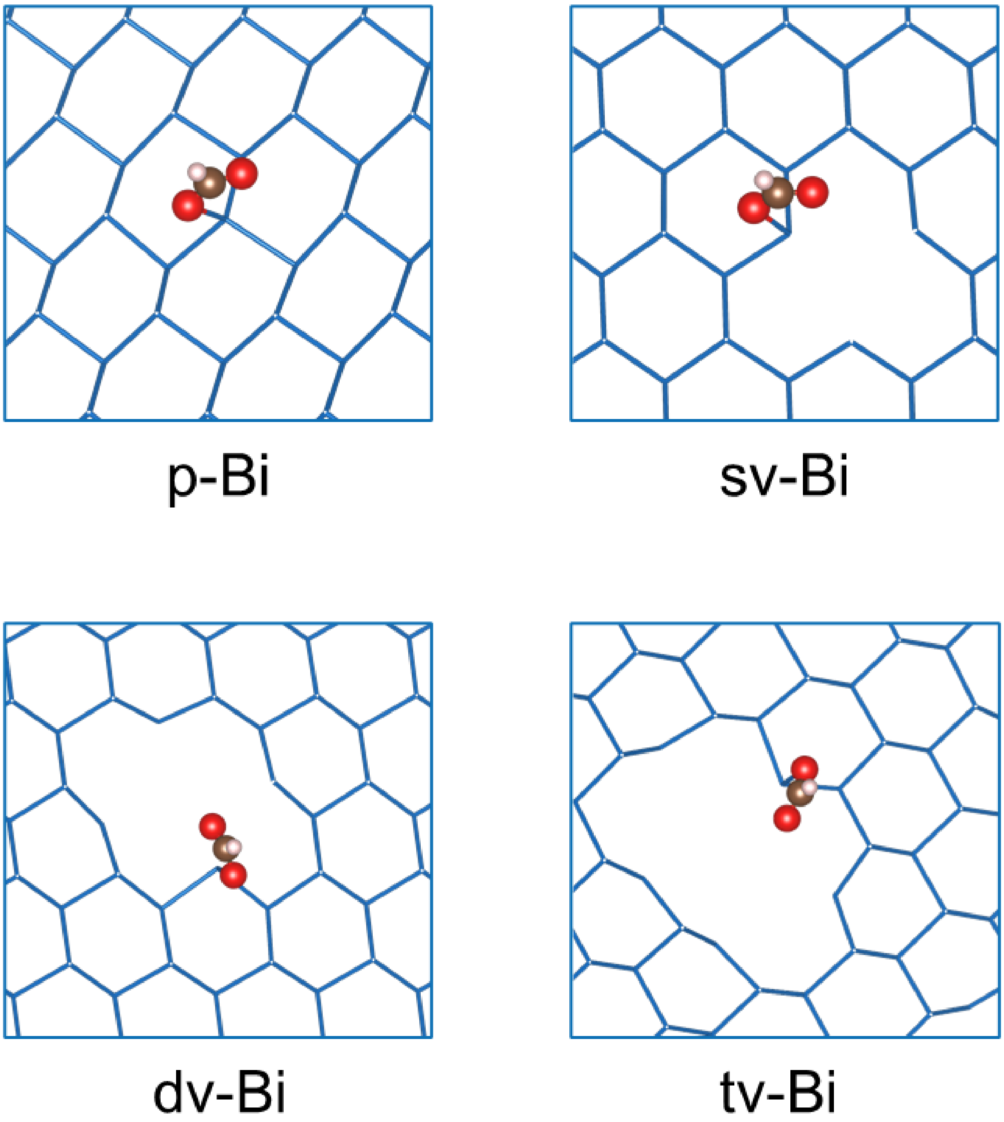

Figure S1. Calculated adsorption models of the *OCHO intermediate for different defect Bi models.

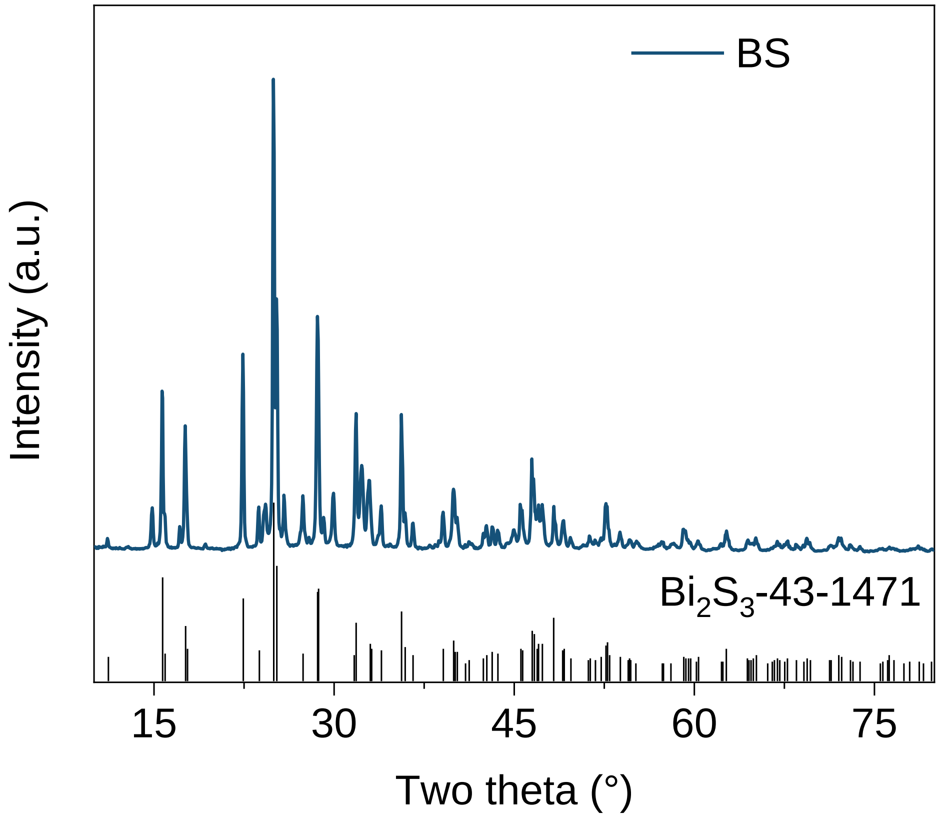

Figure S2. XRD pattern of the Bi_2S_3 (BS) nanowires.

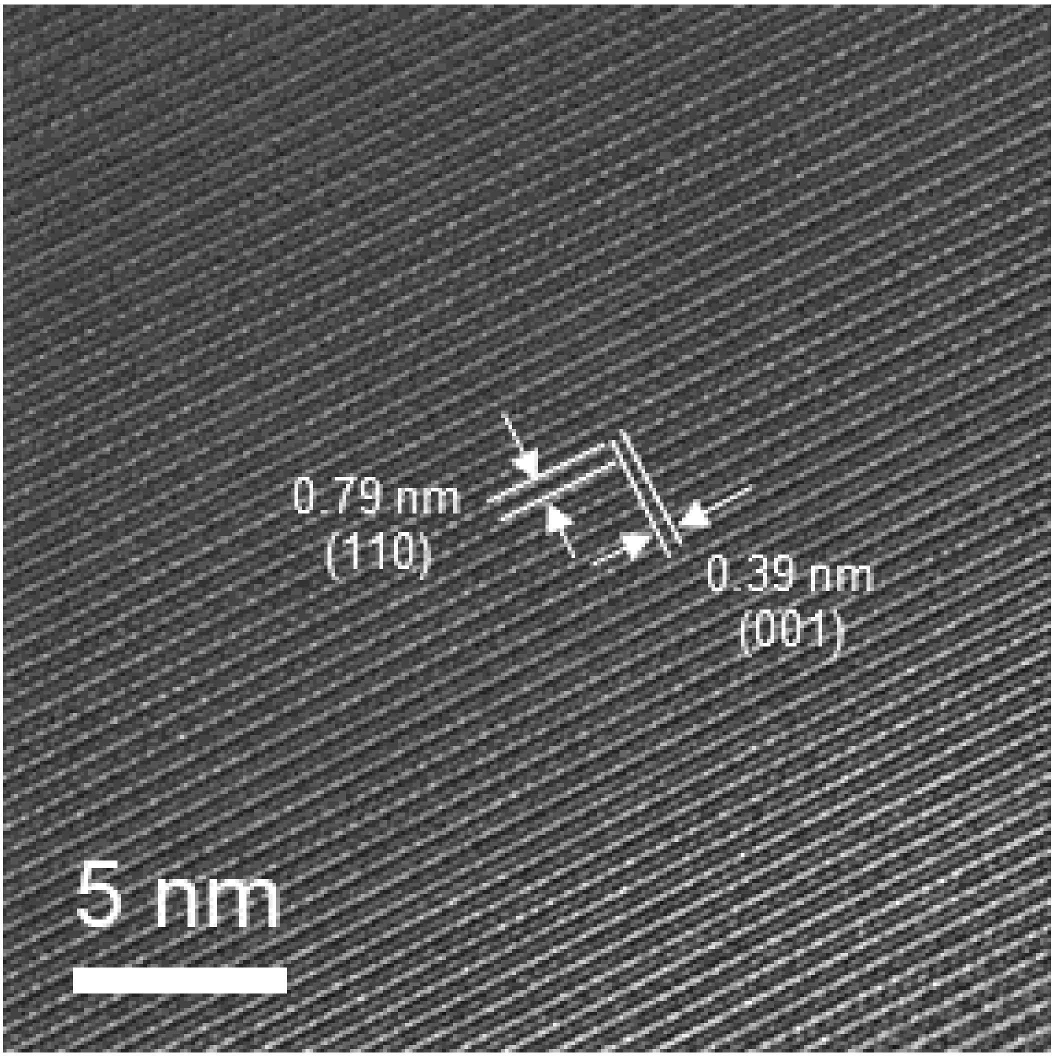

Figure S3. High-resolution transmission electron microscopy (HRTEM) of the BS nanowires.

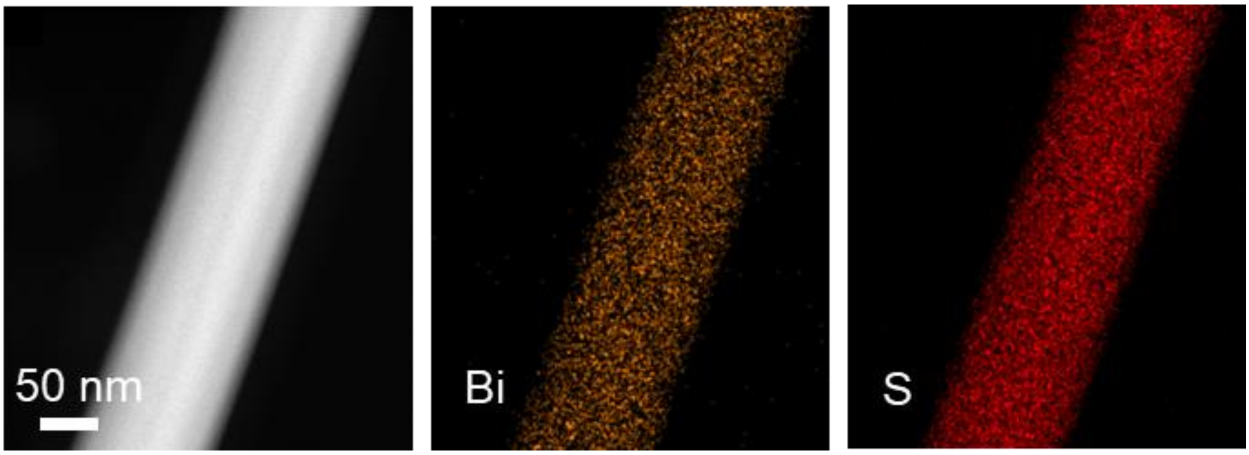

Figure S4. High-angle annular dark-field scanning transmission electron microscopy (HAADF-STEM) image and Energy Dispersive X-ray spectroscopy (EDX) mapping of the BS nanowires.

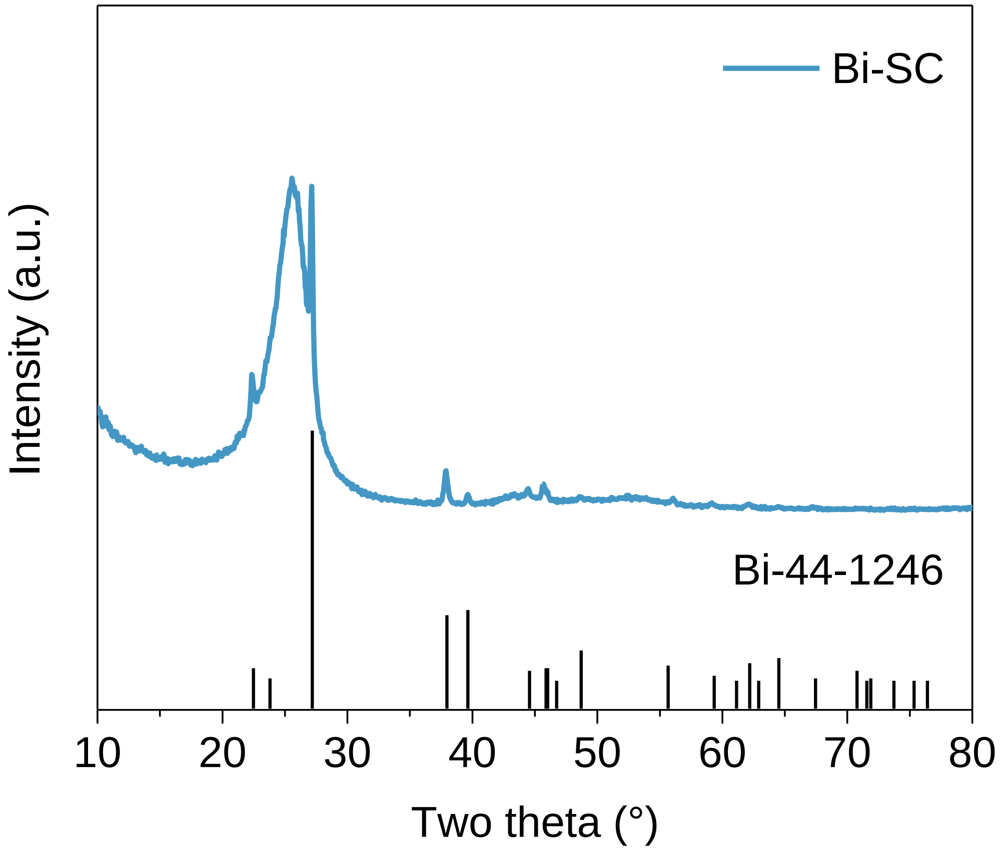

Figure S5. XRD pattern of the sputtered Bi (Bi-SC).

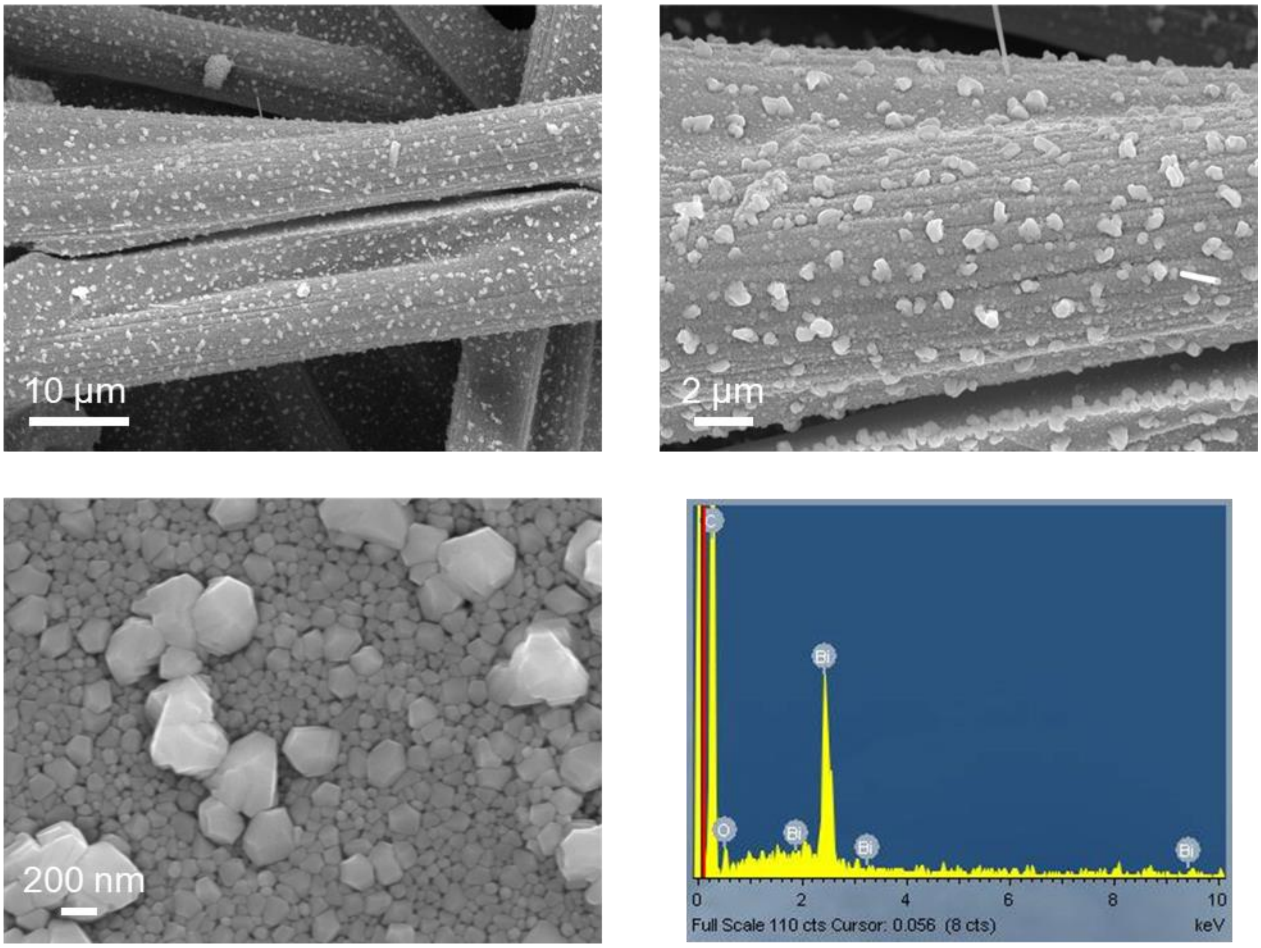

Figure S6. SEM images and Energy Dispersive Spectrum (EDS) of Bi-SC.

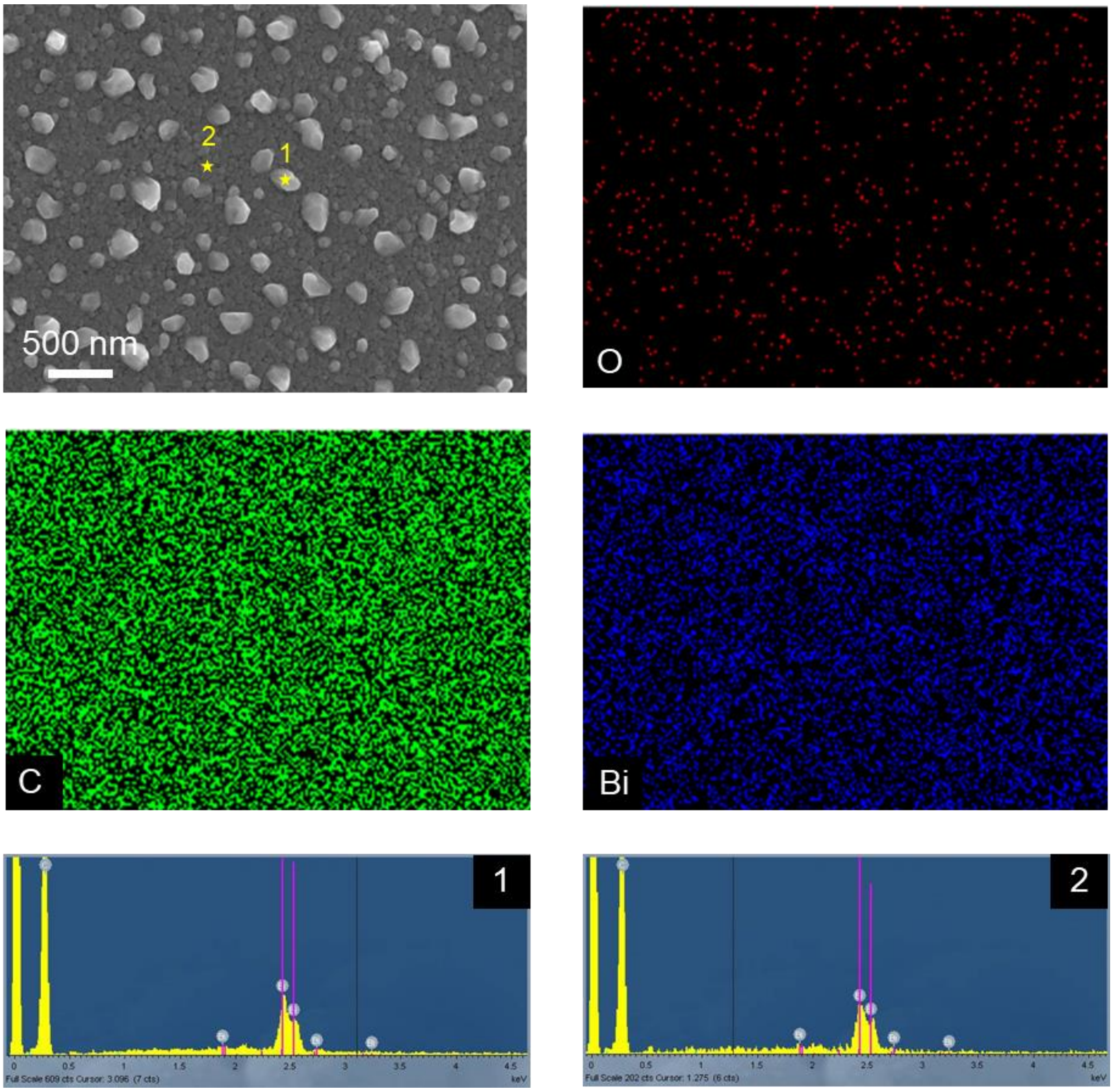

Figure S7. SEM image and corresponding EDS mapping and point signals.

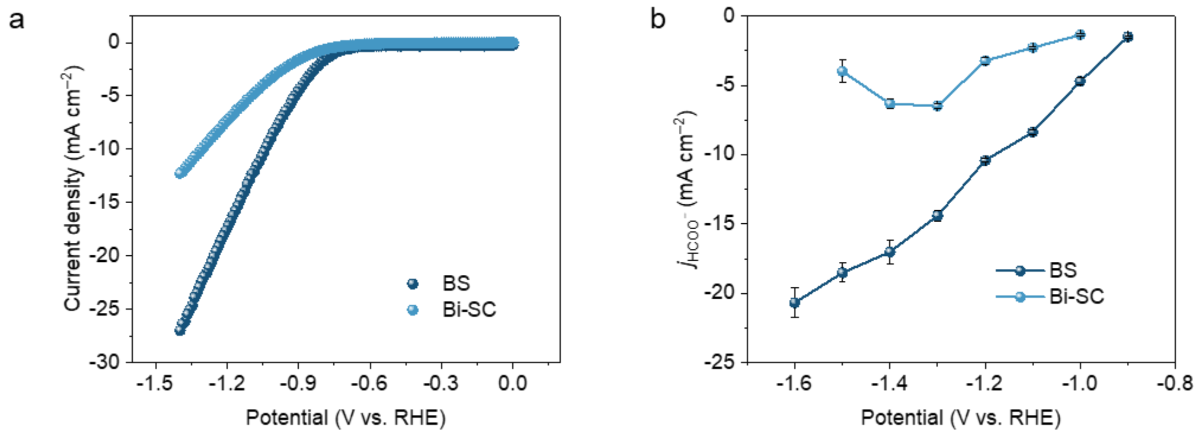

Figure S8. (a) LSV curves of the BS and Bi-SC in 0.5 M $KHCO_3$ electrolyte. (b) Partial current density of $HCOO^-$ under different applied potentials for the BS and Bi-SC. The error bars represent the standard deviation of three measurements.

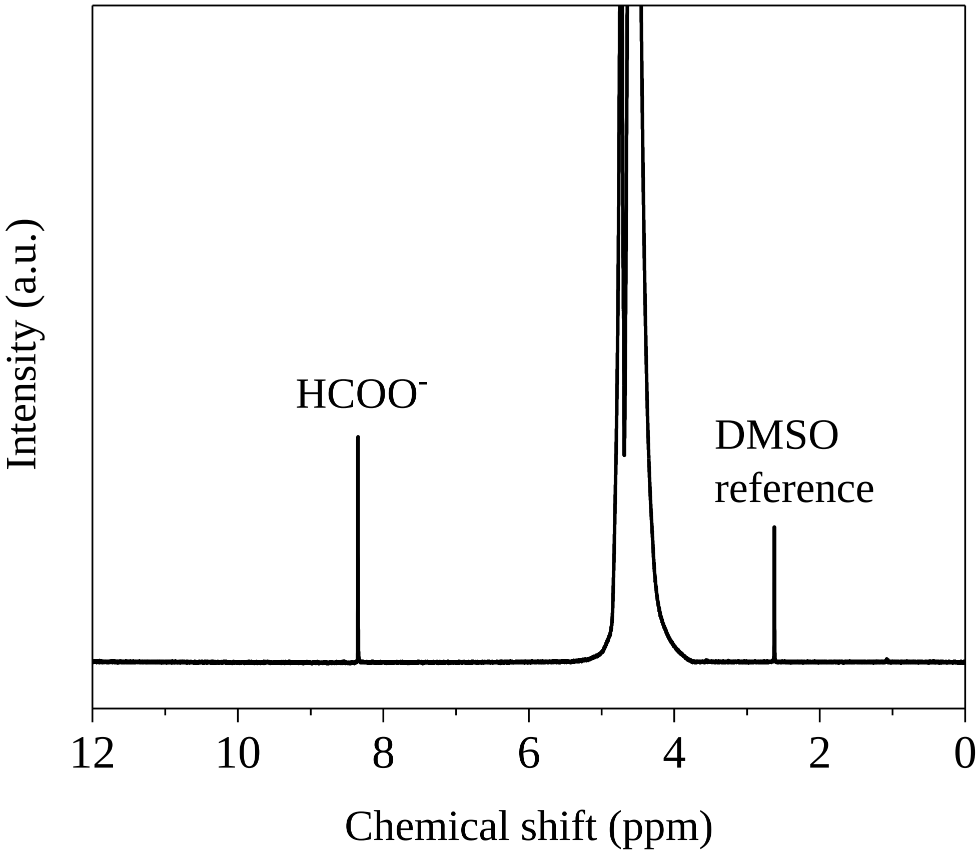

Figure S9. NMR spectrum of the liquid product in H-cell test.

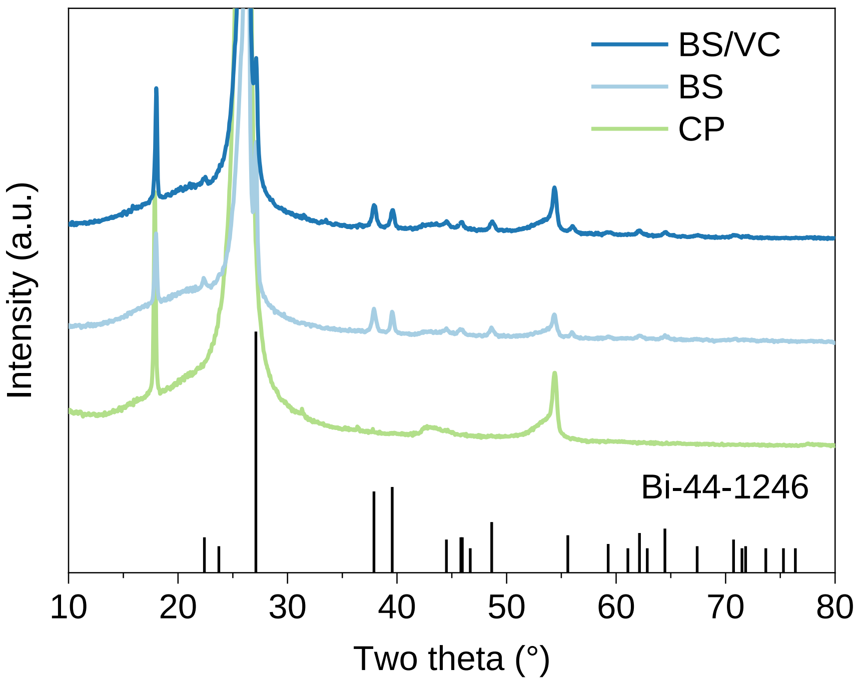

Figure S10. XRD patterns of the BS and BS/VC after the CO_2RR test.

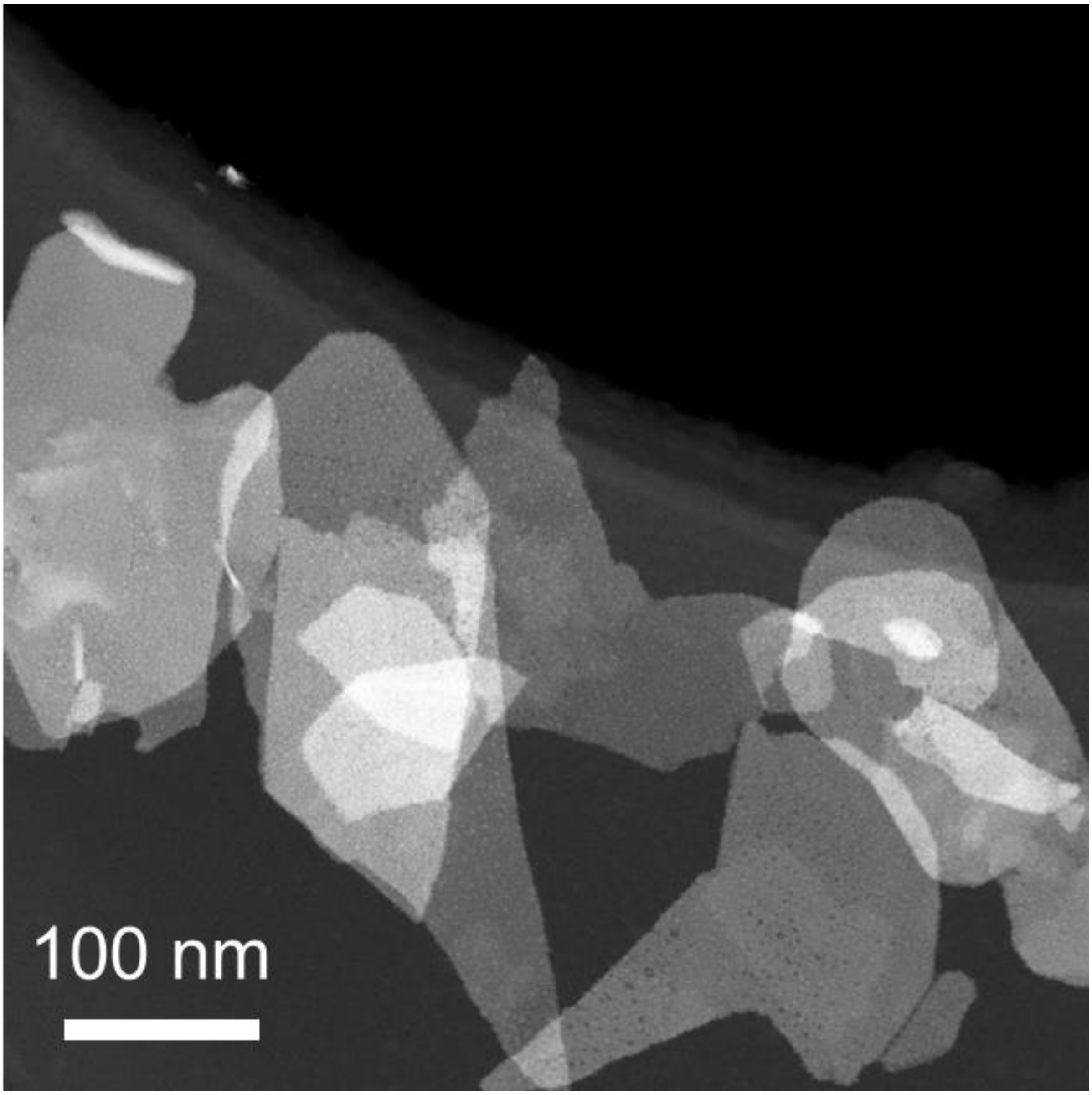

Figure S11. HAADF-STEM image of the BS after the chronoamperometry test.

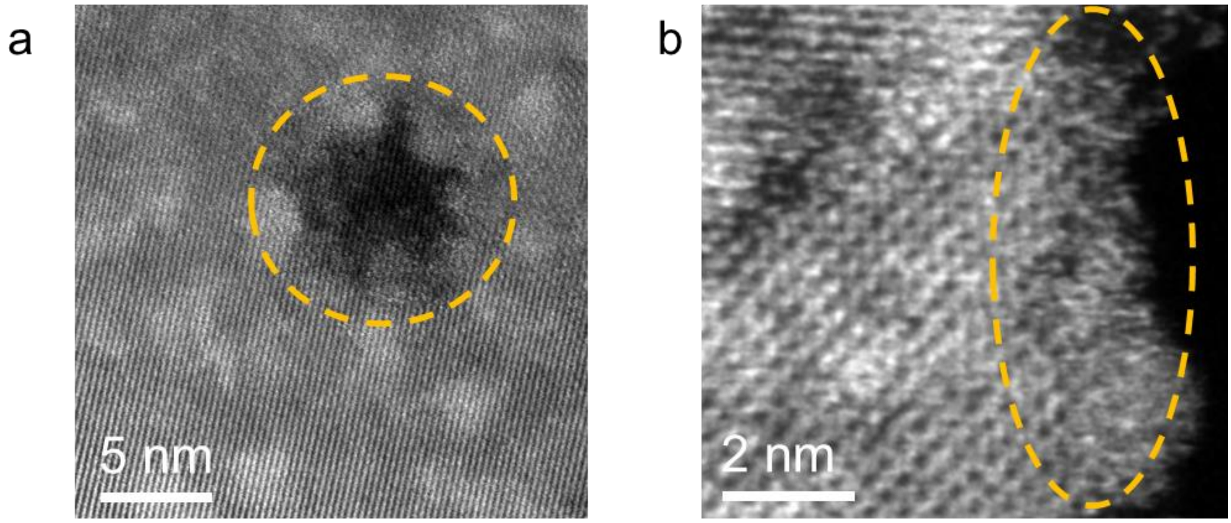

Figure S12. HAADF-STEM images of the BS after the chronoamperometry test.

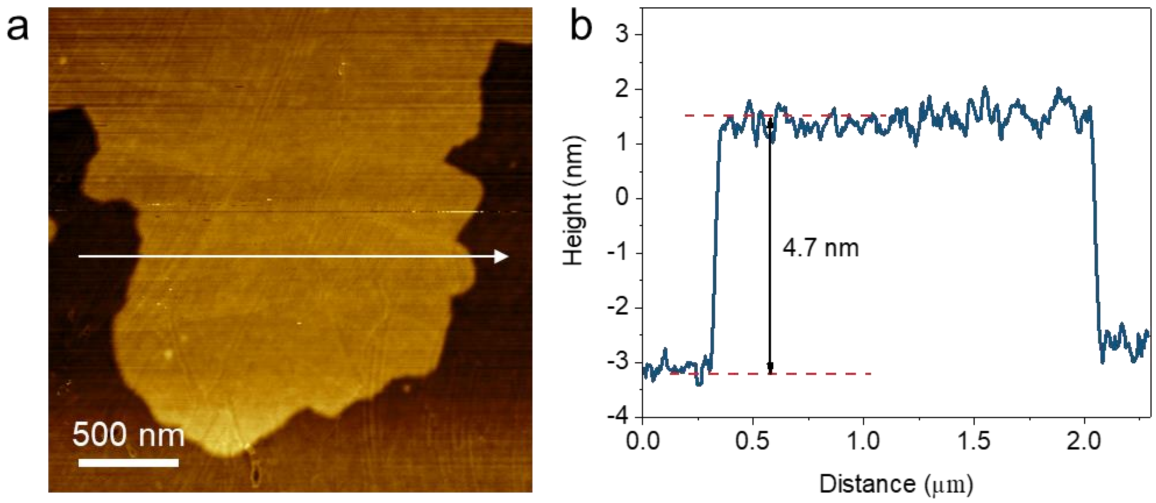

Figure S13. (a) AFM images and (b) corresponding height curves of reconstructed Bi nanosheets. The scanning path for the height analysis is indicated by the white arrow line in (a).

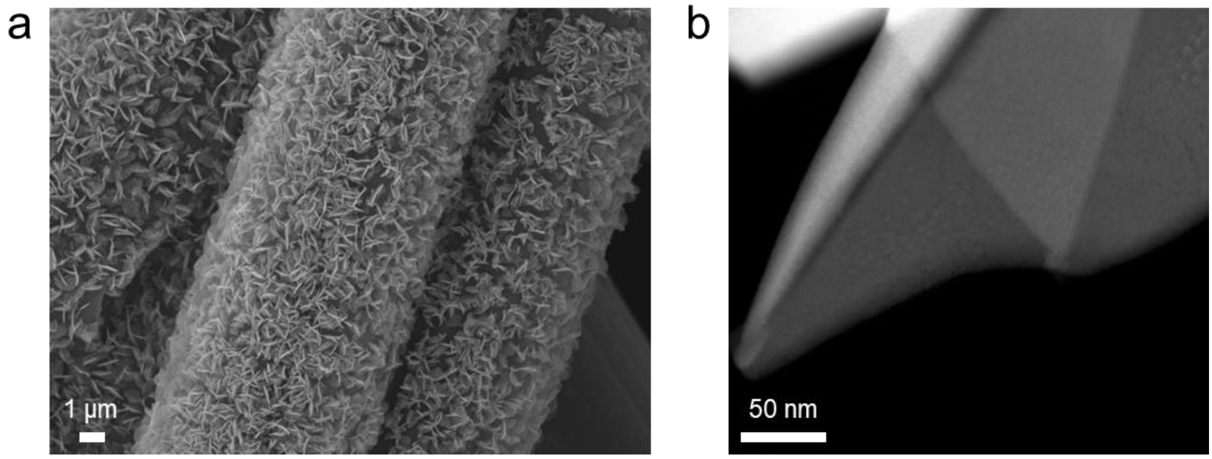

Figure S14. SEM image (a) and HAADF-STEM image (b) of the Bi-SC after the chronoamperometry test.

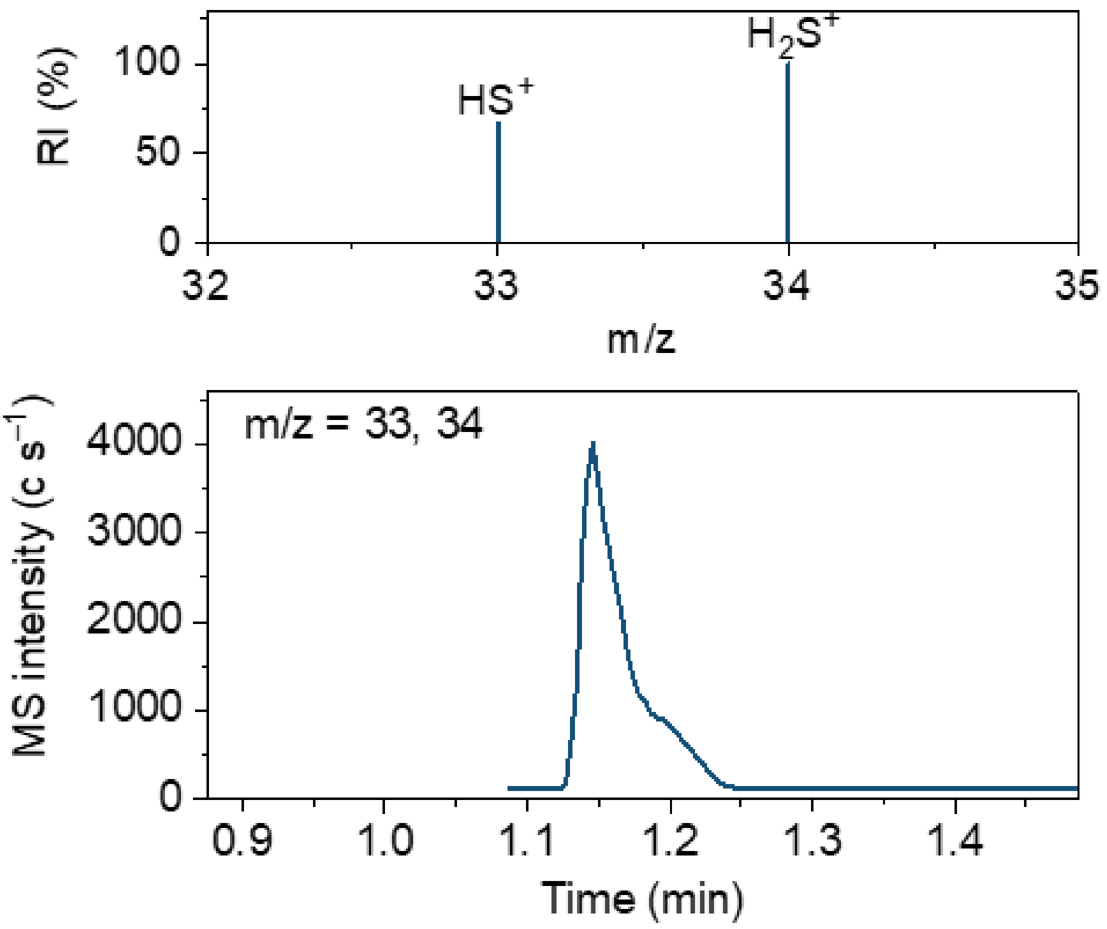

Figure S15. Mass spectrum of BS nanowires at –1.2 V.

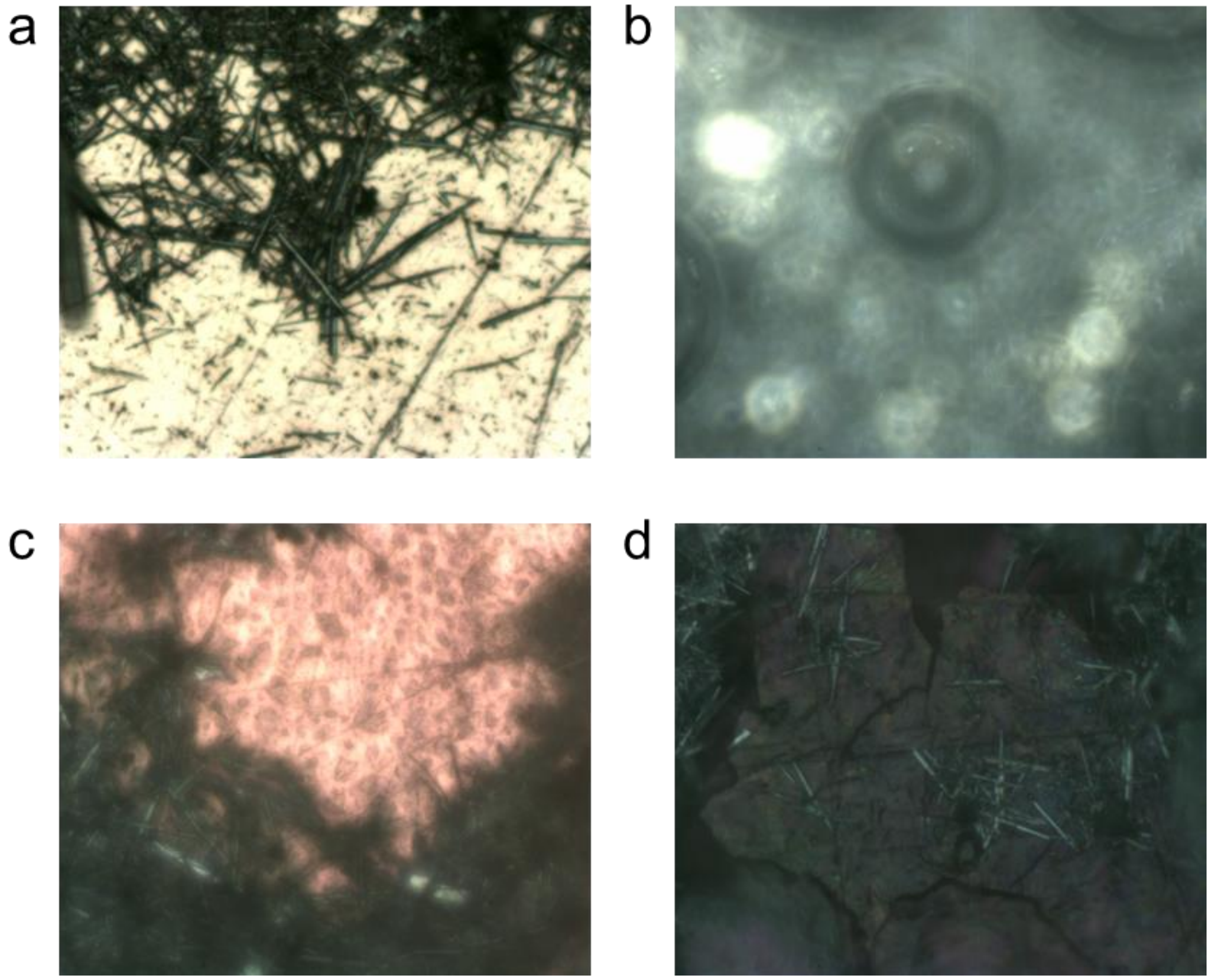

Figure S16. Optical microscope images of the BS nanowires under the open circuit potential (a), applied potential of –1.2 V (b), and removing the bias (c, d).

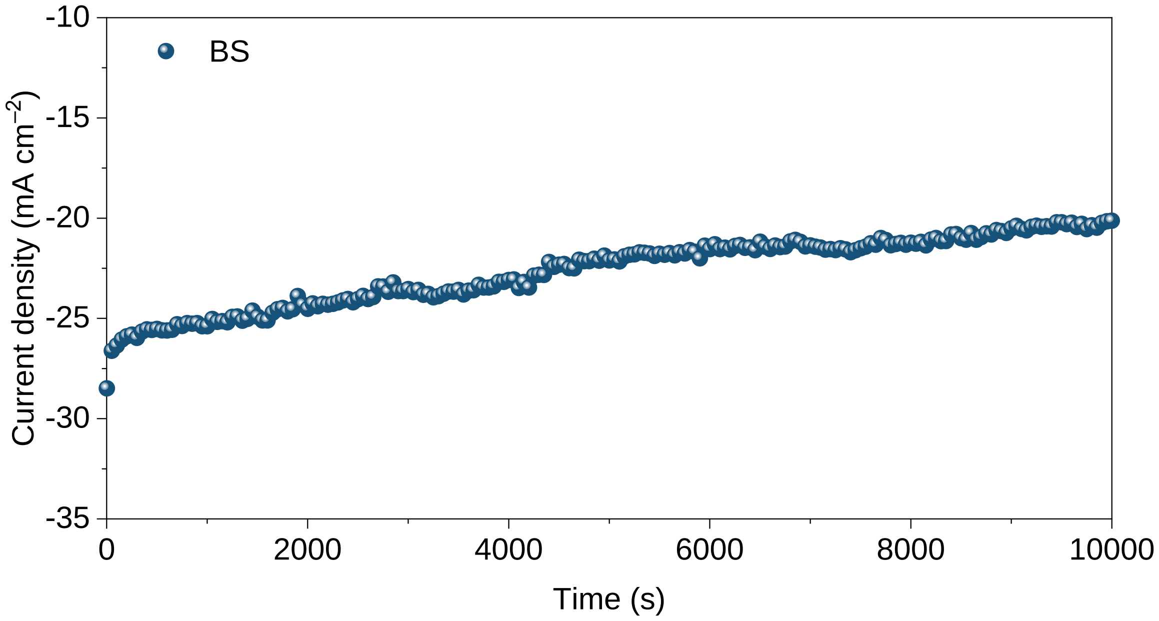

Figure S17. Chronoamperometry test of the BS at –1.1 V in 0.5 M $KHCO_3$.

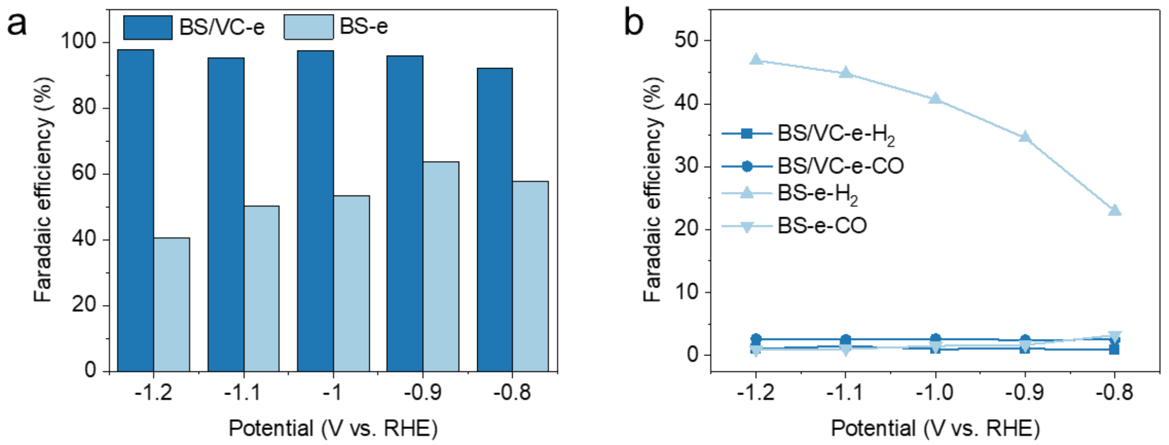

Figure S18. The faradaic efficiency (FE) of $HCOO^{-}$ (a) and H_2, CO (b) at different applied potentials for the BS-derived Bi and BS/VC-derived Bi/VC after exposing in air for 1 h.

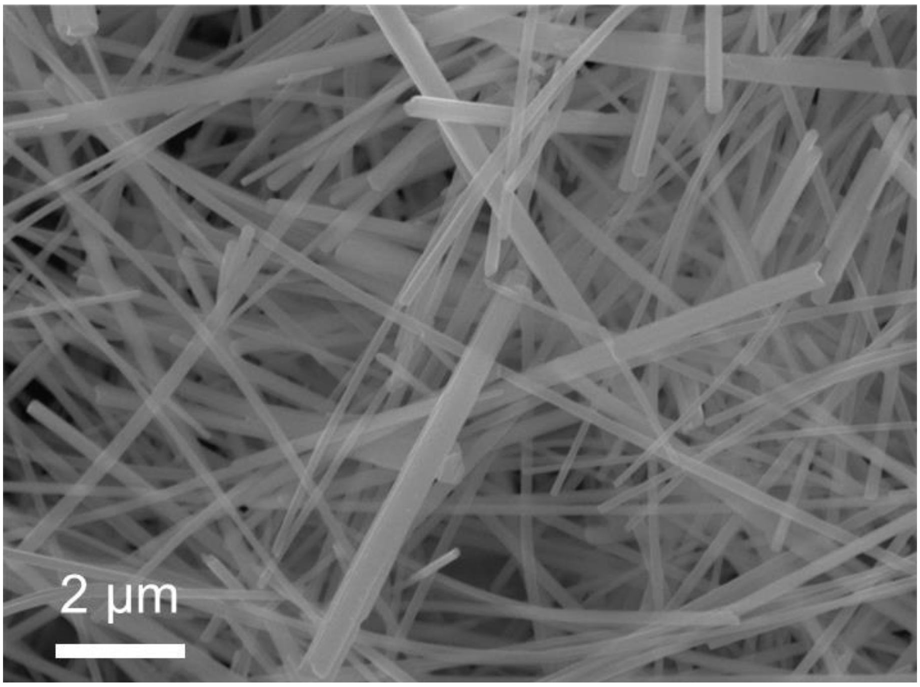

Figure S19. SEM image of the BS/VC.

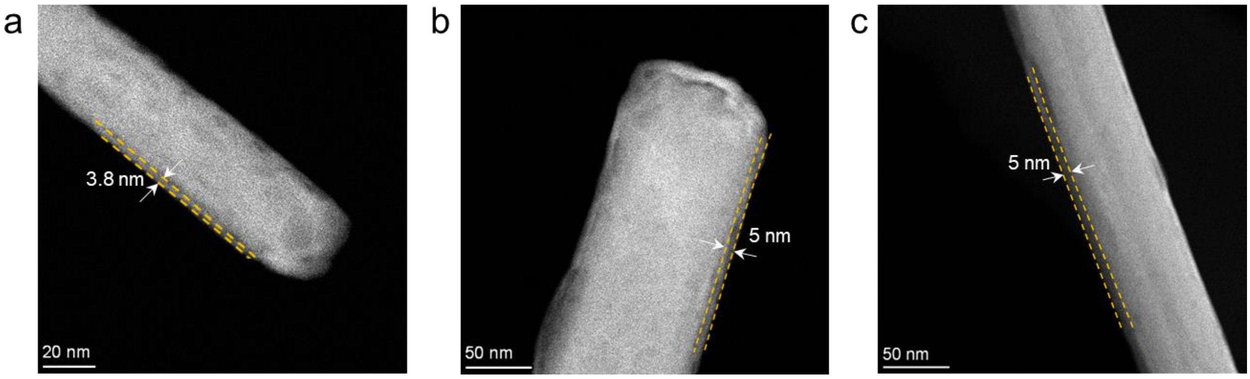

Figure S20. HAADF-STEM image of BS/VC with different VC concentration. (a) 0.25 M, (b) 1 M, (c) 2 M.

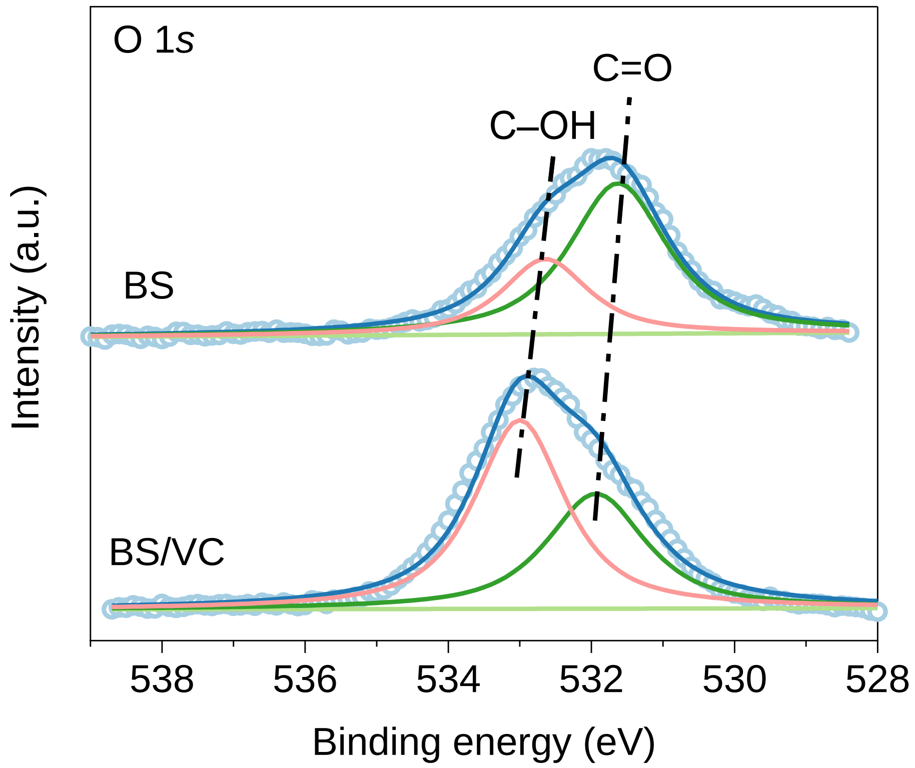

Figure S21. High-resolution O 1*s* spectra for the BS/VC and BS.

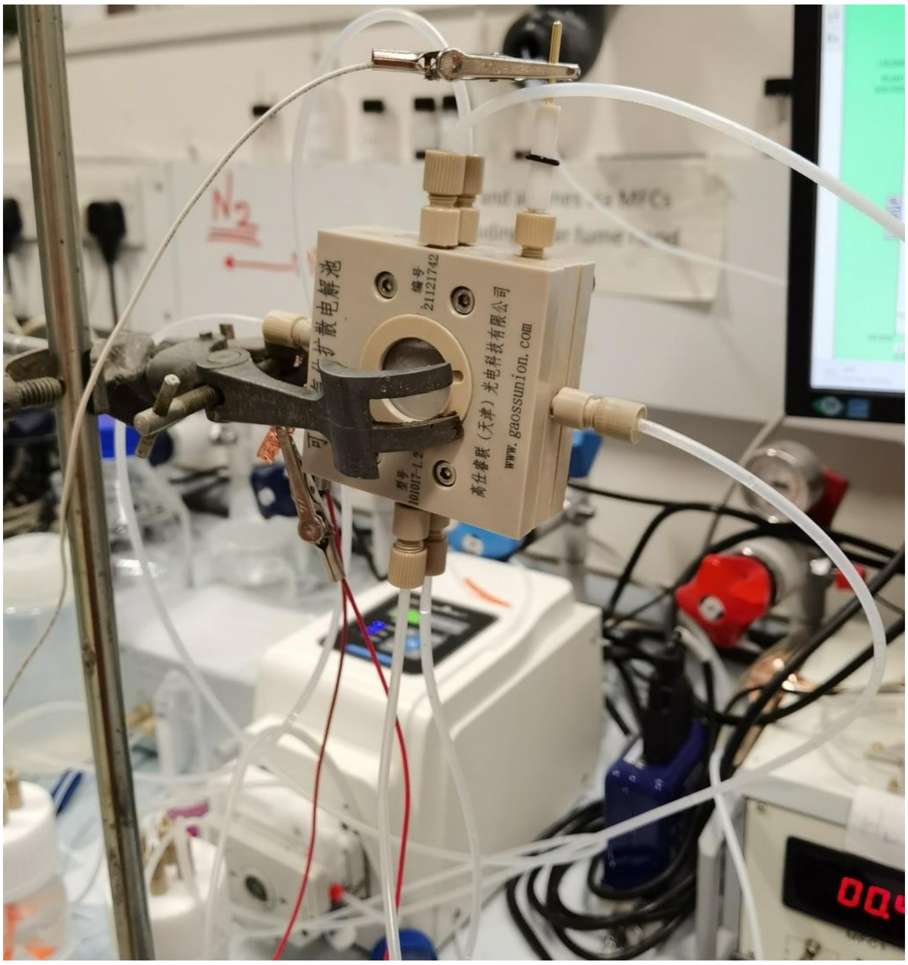

Figure S22. Optical photograph for alkaline flow cell electrolyzer.

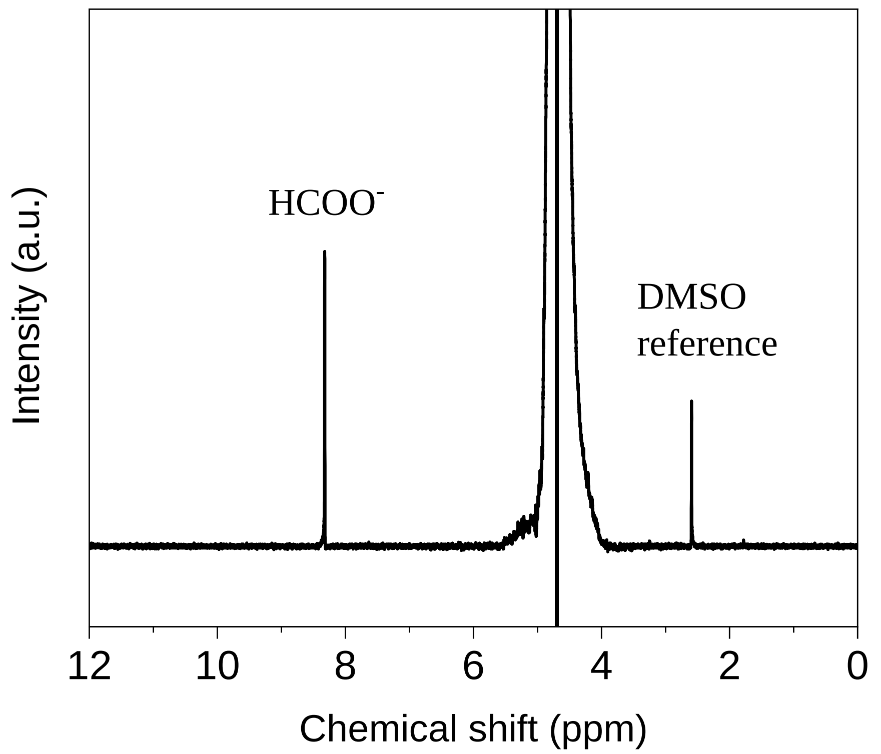

Figure S23. NMR spectrum of the liquid product in alkaline flow-cell system test.

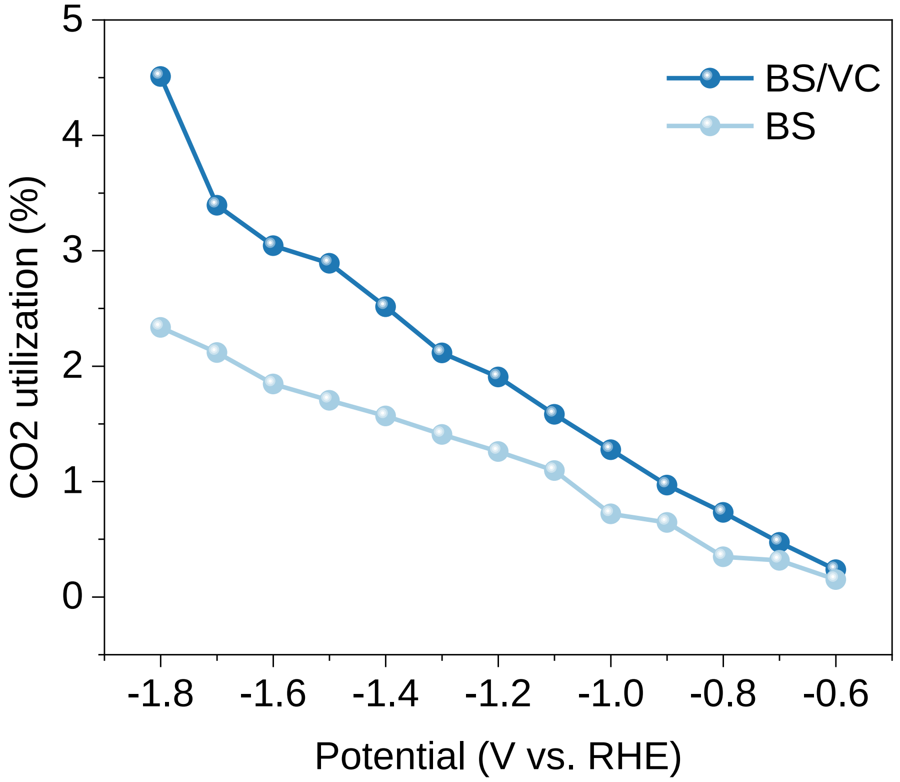

Figure S24. Single pass CO_2 utilization efficiency of BS and BS/VC in alkaline flow-cell system.

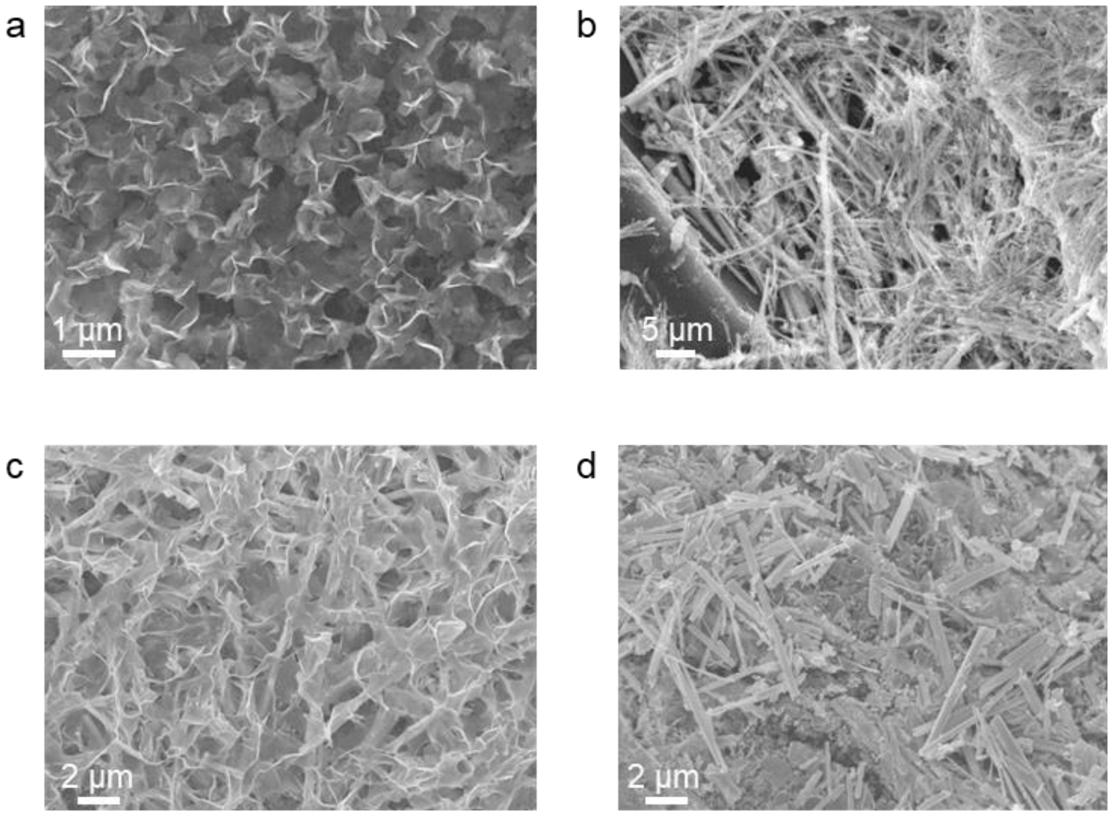

Figure S25. SEM images of the (a) BS-derived Bi and (b) BS/VC-derived Bi/VC on carbon paper after 24 hours electrolysis in 0.5 M $KHCO_3$ at 30 mA cm^{-2}. SEM images of the (c) BS-derived Bi and (d) BS/VC-derived Bi/VC on gas diffusion electrode after 24 hours electrolysis in 1 M KOH at 200 mA cm^{-2}.

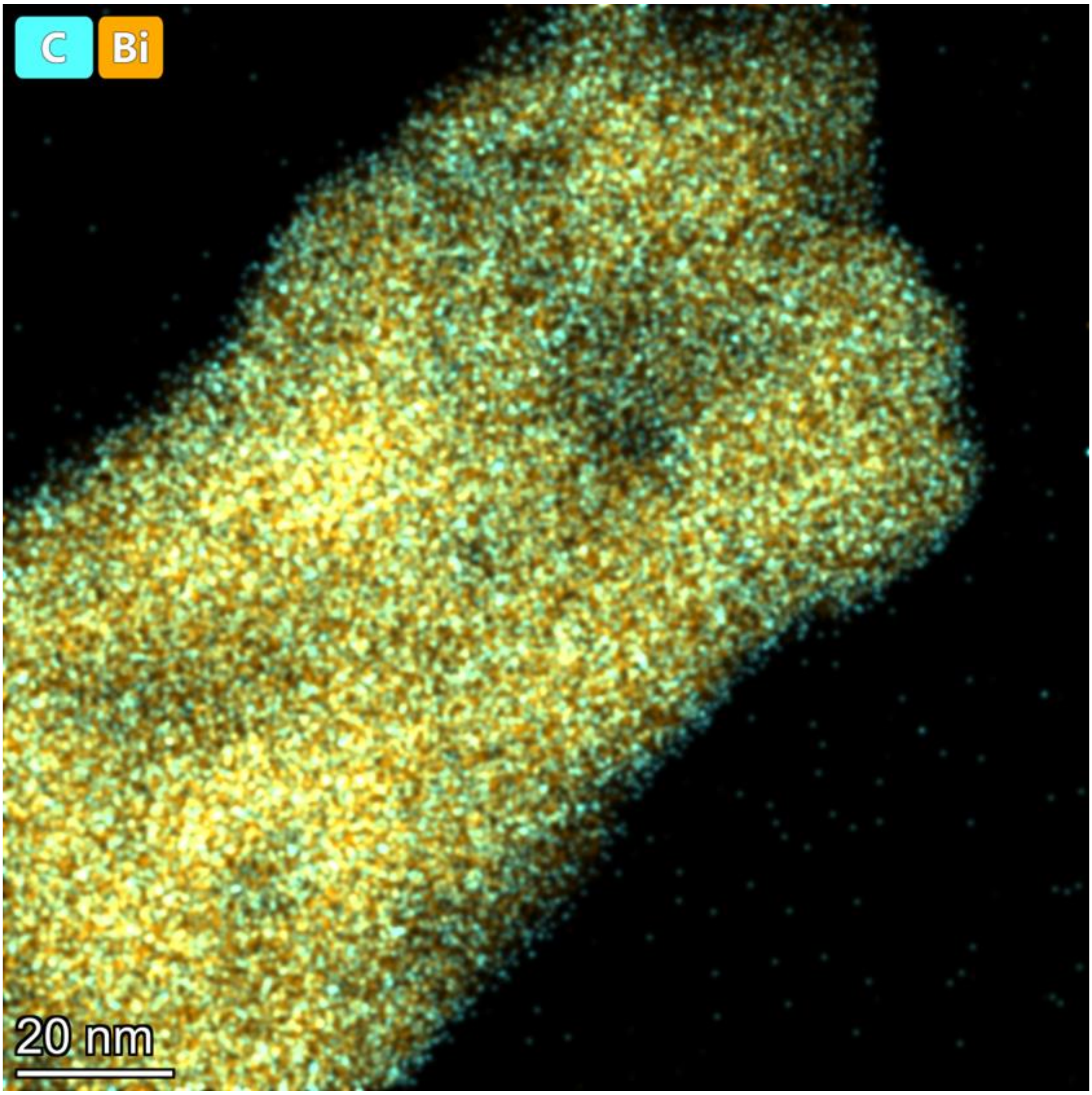

Figure S26. EDS mapping of Bi and C element in the BS/VC-derived Bi/VC nanowires.

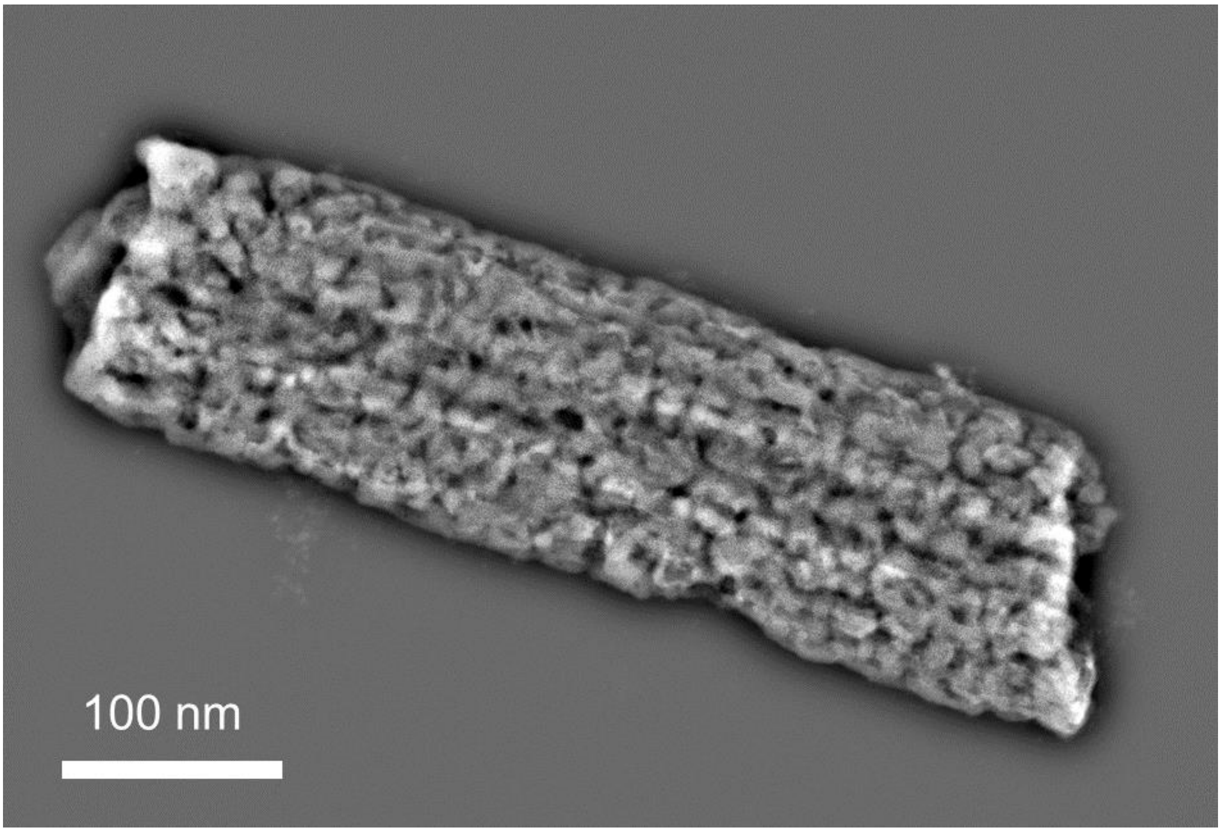

Figure S27. High pass filtered HAADF-STEM image of the BS/VC-derived Bi/VC nanowire.

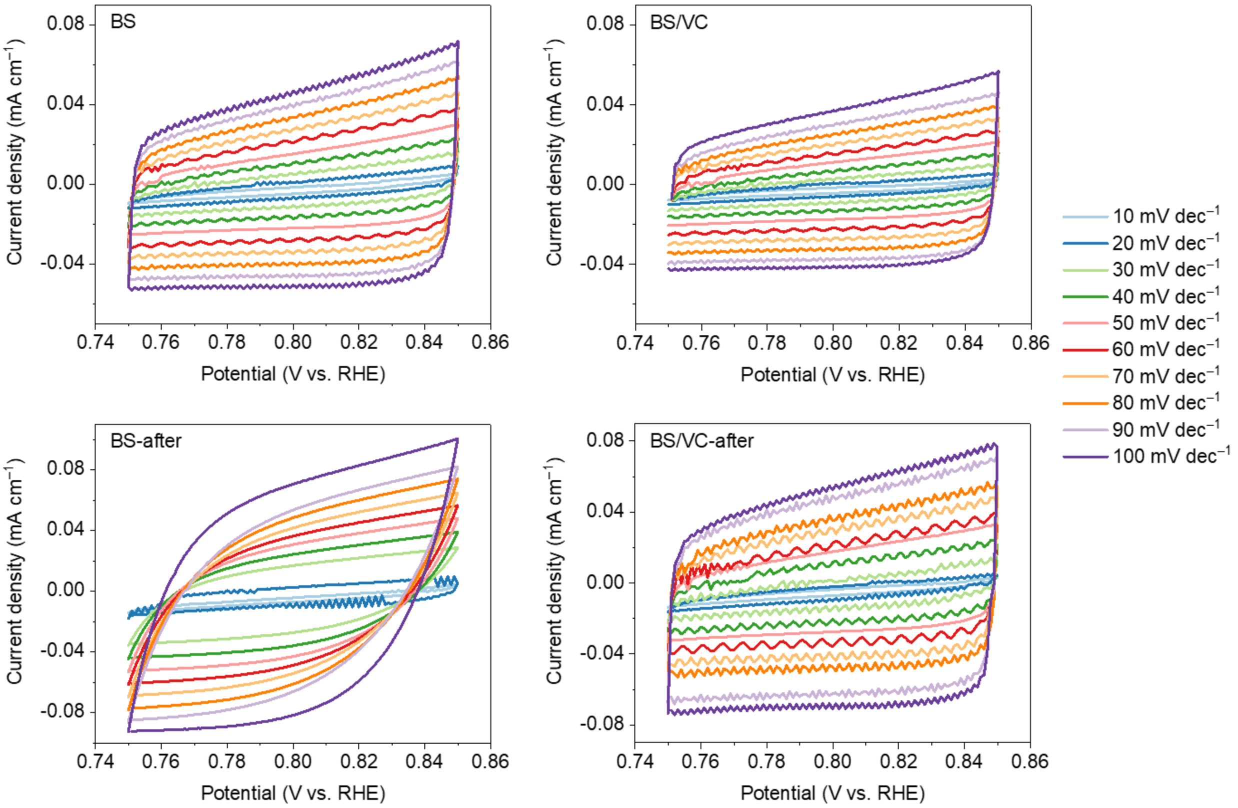

Figure S28. The CV curves with different scan rate under the potential of 0.75 V – 0.85 V of BS, BS/VC, BS-after, and BS/VC-after. To avoid the affect of porous electrode (gas diffusion electrode) and electrolyte flow, we conducted the electrochemical surface area (ECSA) test by dropping the catalysts ink on Indium Tin Oxide (ITO) coated glass and run the CV in 1 M KOH without flow.

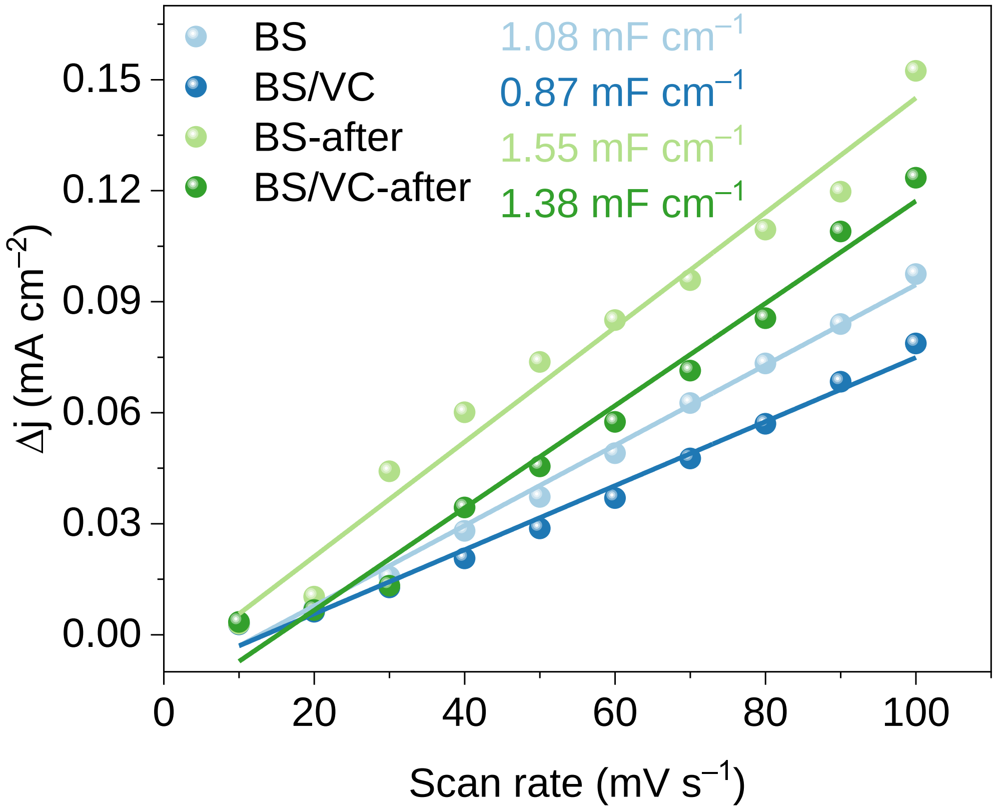

Figure S29. Δ*j* at 0.8 V vs. RHE as a function of the scan rate to evaluate C_{dl}.

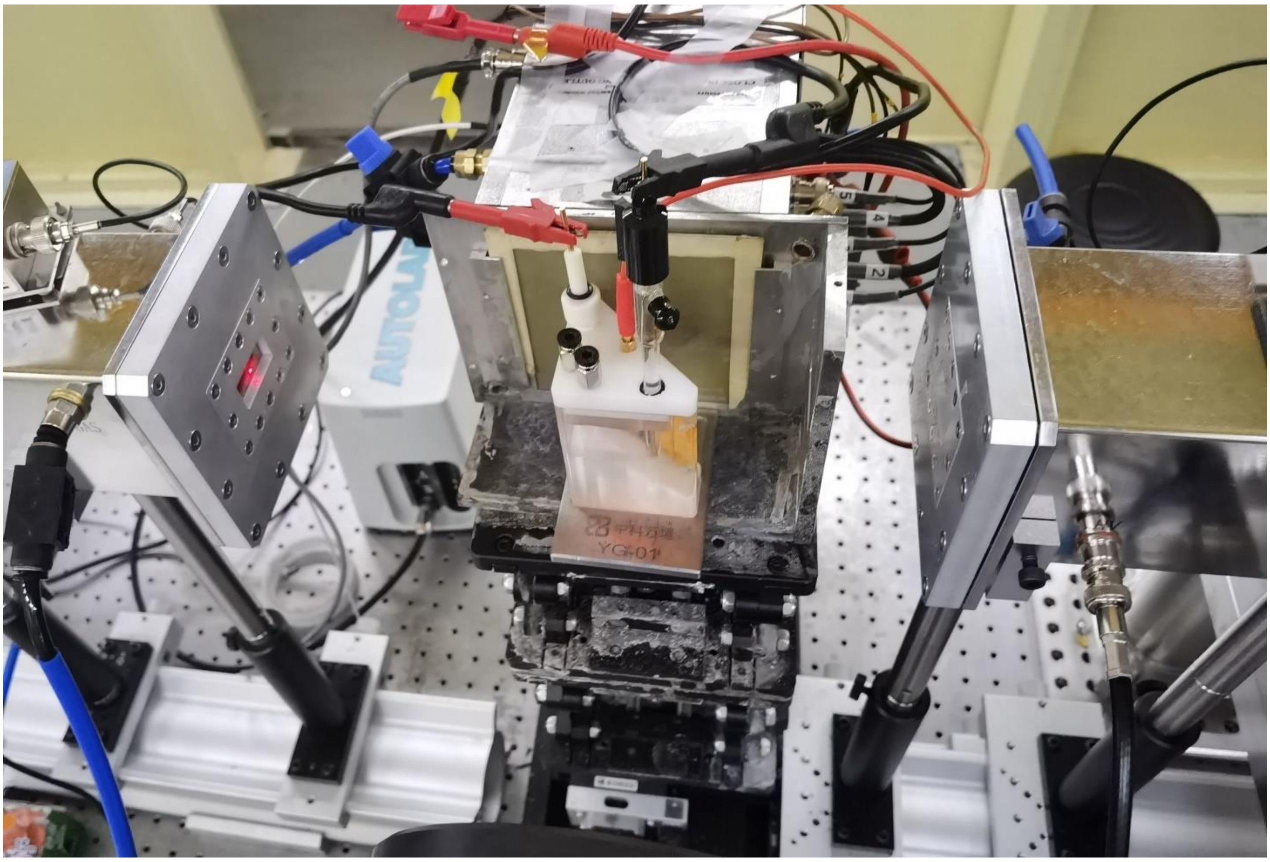

Figure S30. Optical photograph for in-situ XAFS measurements.

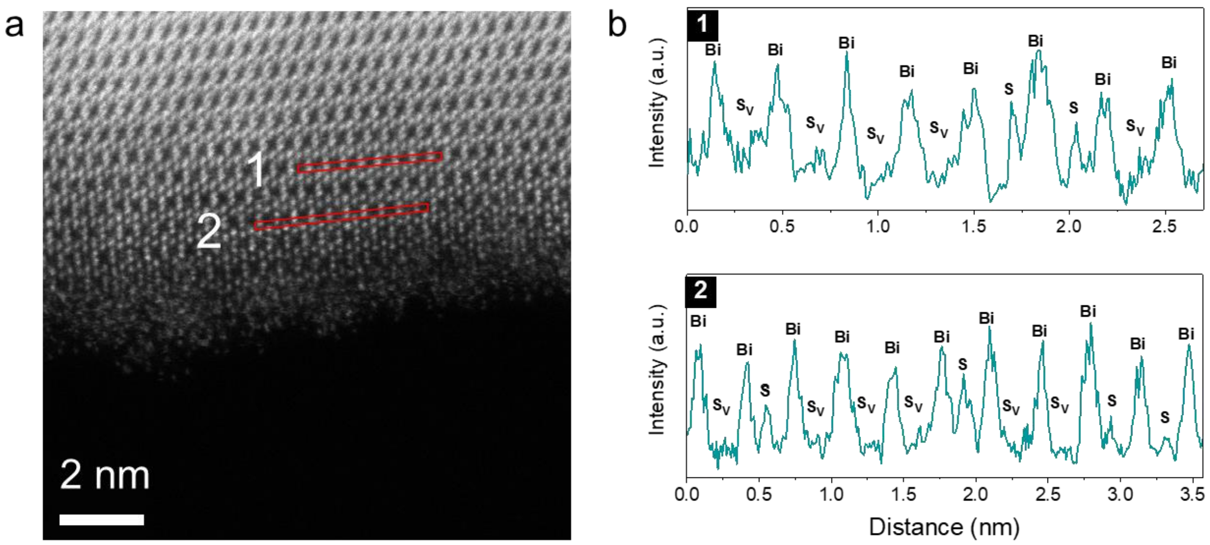

Figure S31. HAADF-STEM image (a) and line scan intensity signal (b) of BS/VC.

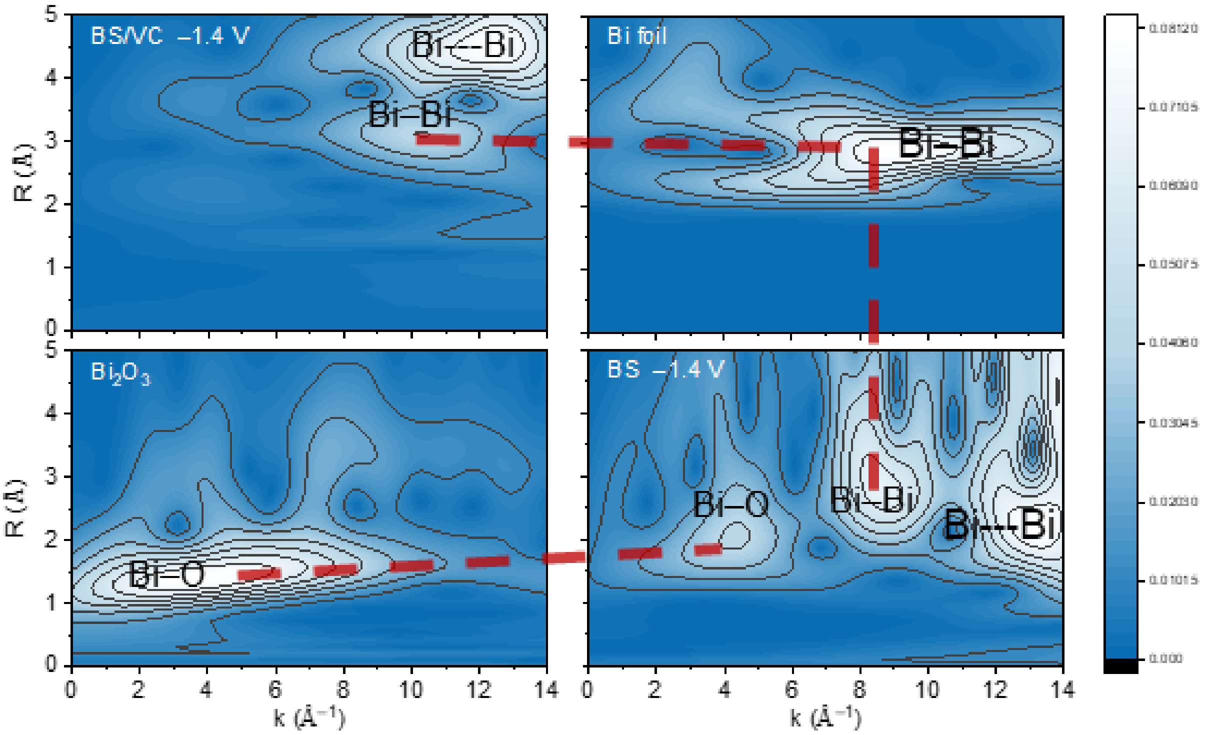

Figure S32. Wavelet transform images for the Bi L_3-edge signals for the BS and BS/VC at –1.4 V. Bulk Bi foil and Bi_2O_3 are listed as references.

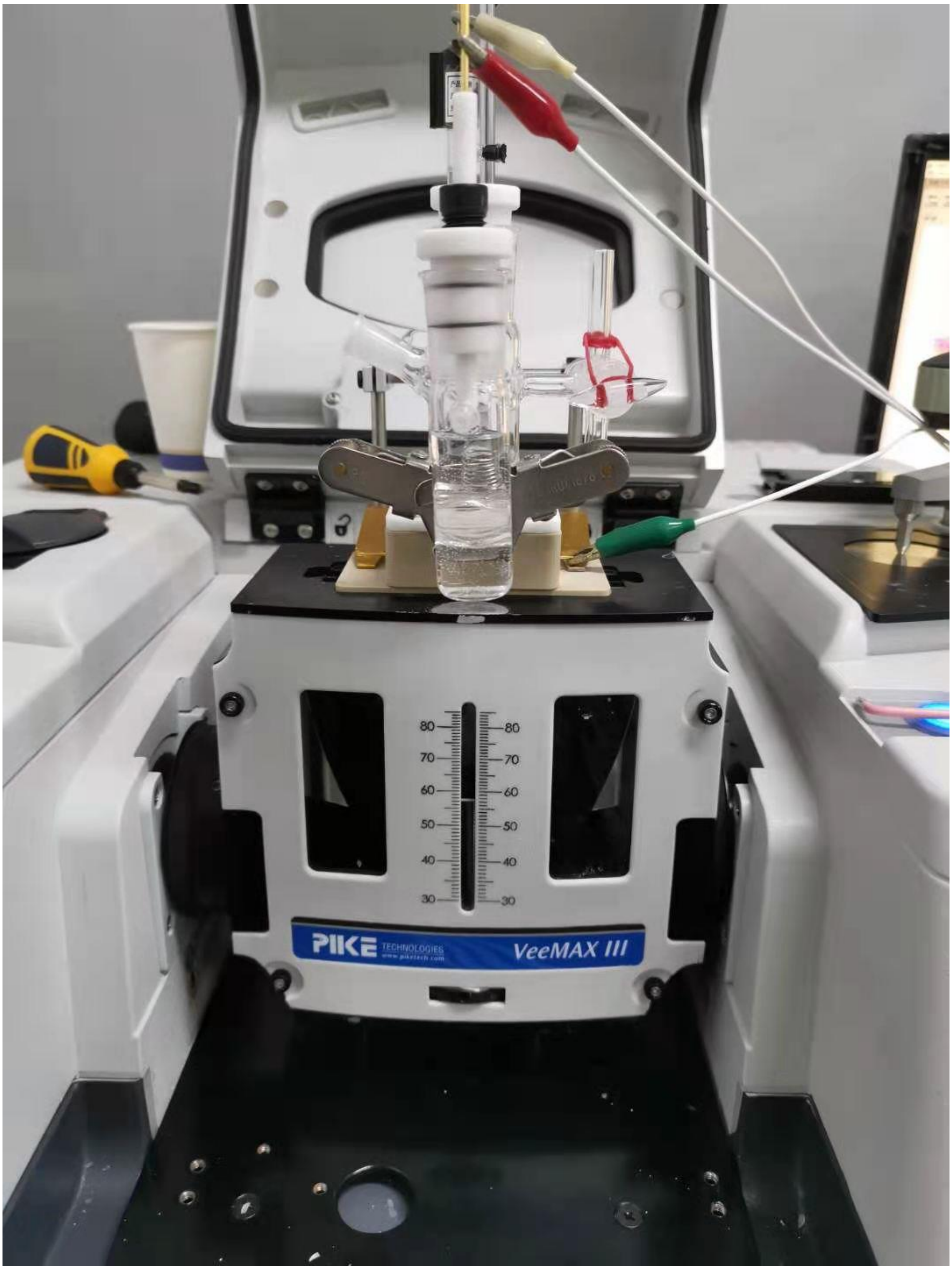

Figure S33. Schematic and optical photographs of in-situ attenuated total reflection surface enhanced infrared adsorption spectroscopy (ATR-SEIRAS).

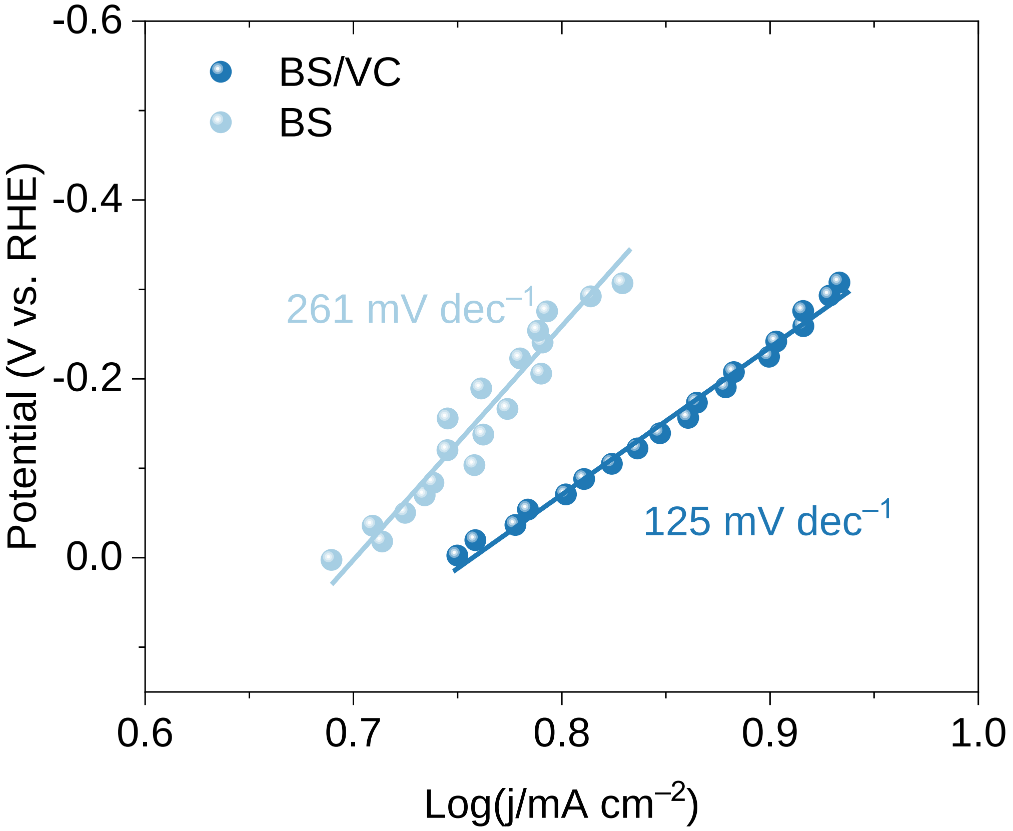

Figure S34. Tafel slope of BS and BS/VC.

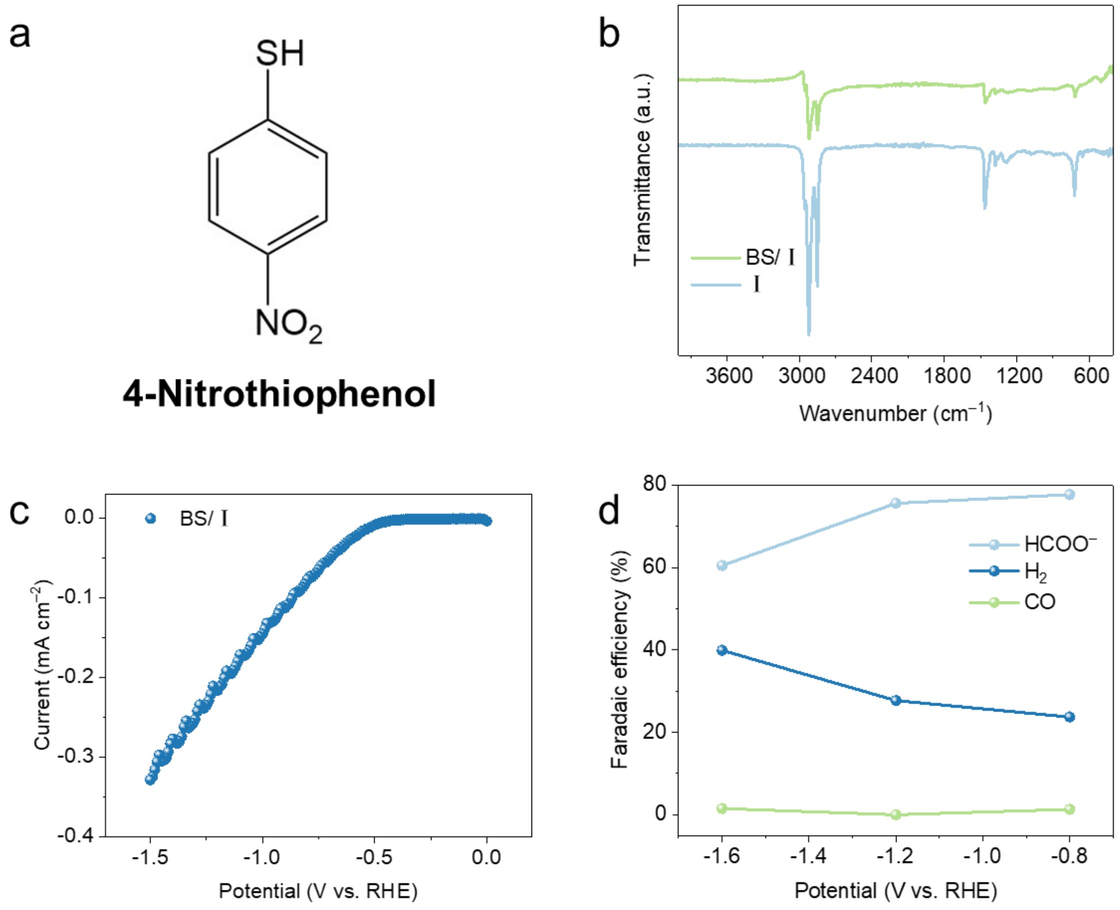

Figure S35. (a) The structure formula of 4-Nitrothiophenol. (b) FT-IR analysis of BS/**I**, and **I**. **I** represent the 4-Nitrothiophenol. (c) The LSV curve of BS/**I** in flow-cell system with 1 M KOH electrolyte. (d) The selectivity of $HCOO^{-}$, H_2, and CO under different applied potential.

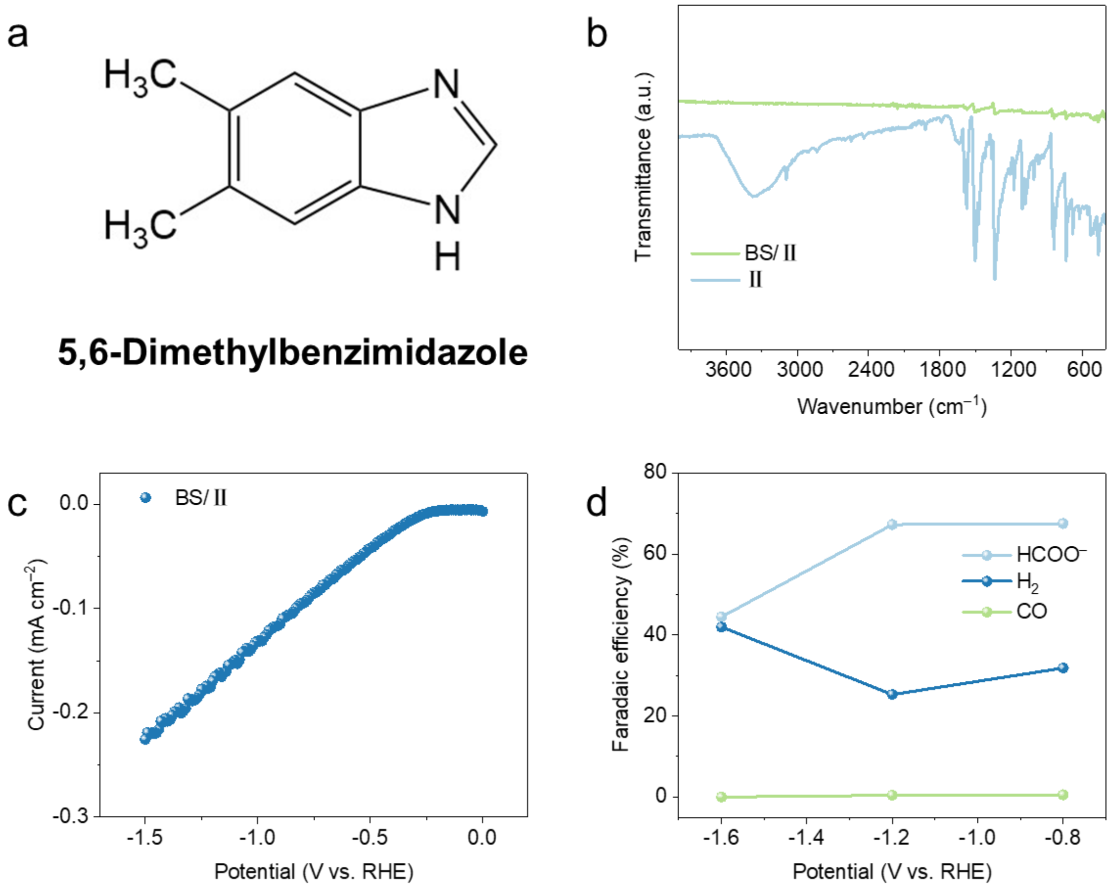

Figure S36. (a) The structure formula of 5,6-Dimethylbenzimidazole. (b) FT-IR analysis of BS/**II**, and **II**. **II** represent the 5,6-Dimethylbenzimidazole. (c) The LSV curve of BS/**II** in flow-cell system with 1 M KOH electrolyte. (d) The selectivity of $HCOO^-$, H_2, and CO under different applied potential.

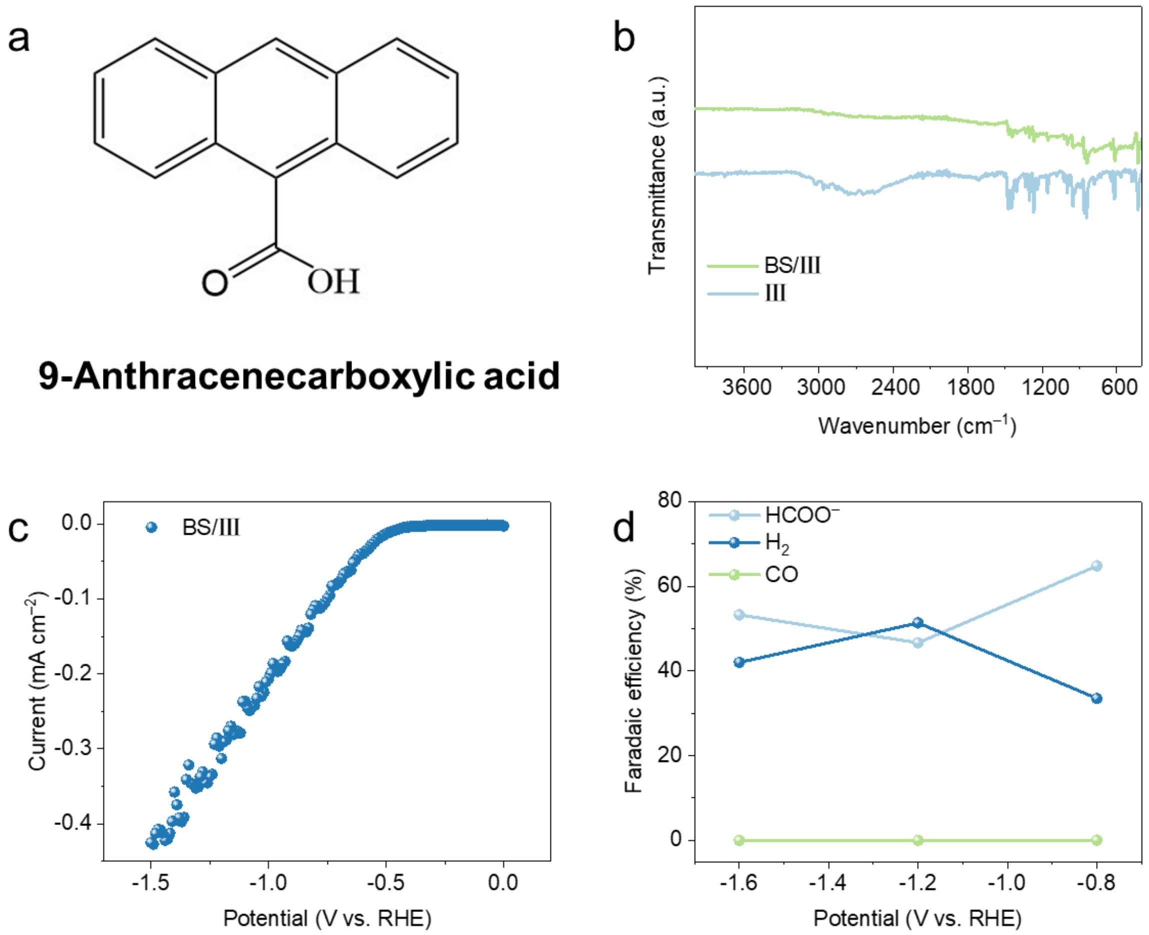

Figure S37. (a) The structure formula of 9-Anthracenecarboxylic acid. (b) FT-IR analysis of BS/**III**, and **III**. **III** represent the 9-Anthracenecarboxylic acid. (c) The LSV curve of BS/**III** in flow-cell system with 1 M KOH electrolyte. (d) The selectivity of $HCOO^{-}$, H_2, and CO under different applied potential.

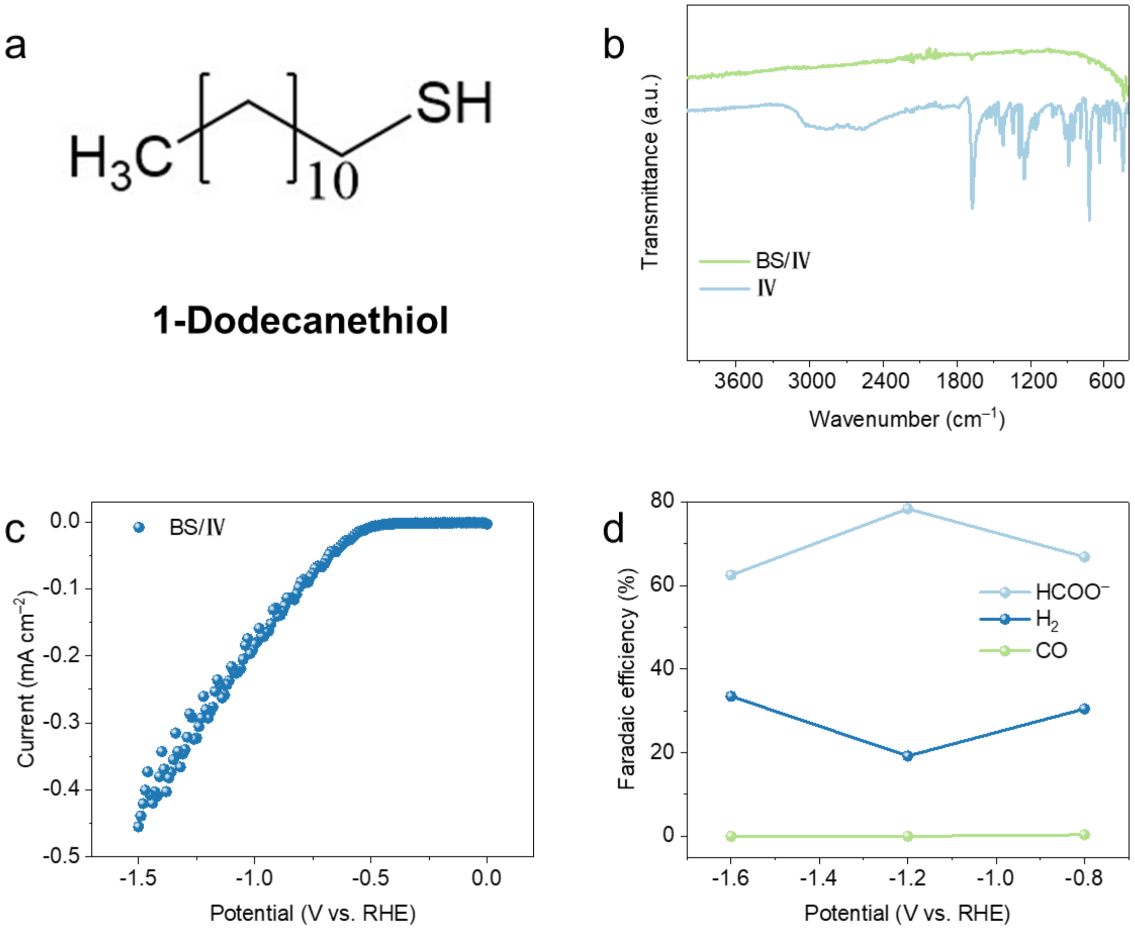

Figure S38. (a) The structure formula of 1-Dodecanethiol. (b) FT-IR analysis of BS/**IV**, and **IV**. **IV** represent the 1-Dodecanethiol. (c) The LSV curve of BS/**IV** in flow-cell system with 1 M KOH electrolyte. (d) The selectivity of $HCOO^-$, H_2, and CO under different applied potential.

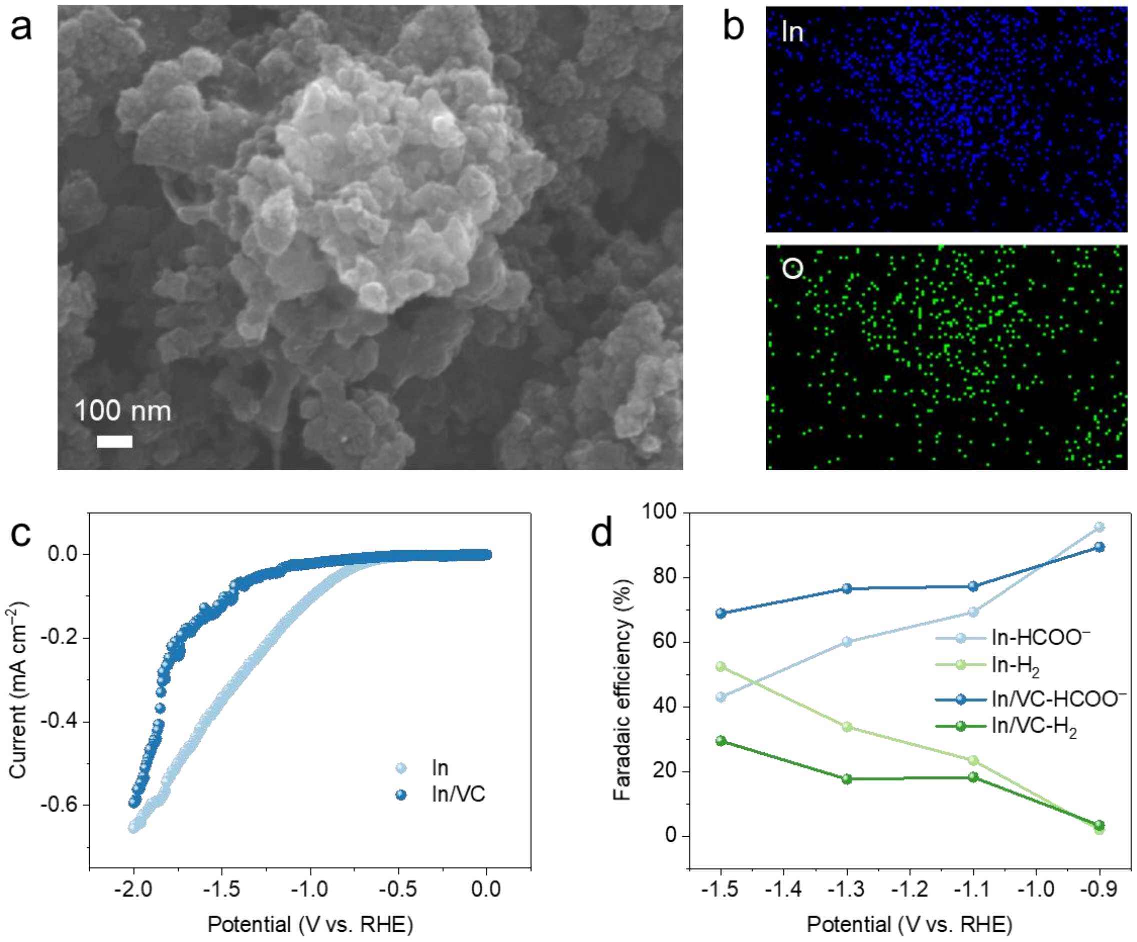

Figure S39. (a) SEM image of In/VC catalyst. In electrode was synthesized by sputtering metal In target on the gas diffusion electrode using a magnetron sputtering machine. The sputtering process was carried out at a power of 1 W for 10 min. In/VC was obtained by spraying VC dispersion solution on In electrode and dried in vacuum oven for 10 hours. (b) EDS mapping of In and O elements in In/VC electrode. (c) The LSV curves of In and In/VC electrode in alkaline flow-cell system with 1 M KOH. (d) The corresponding products of In and In/VC.

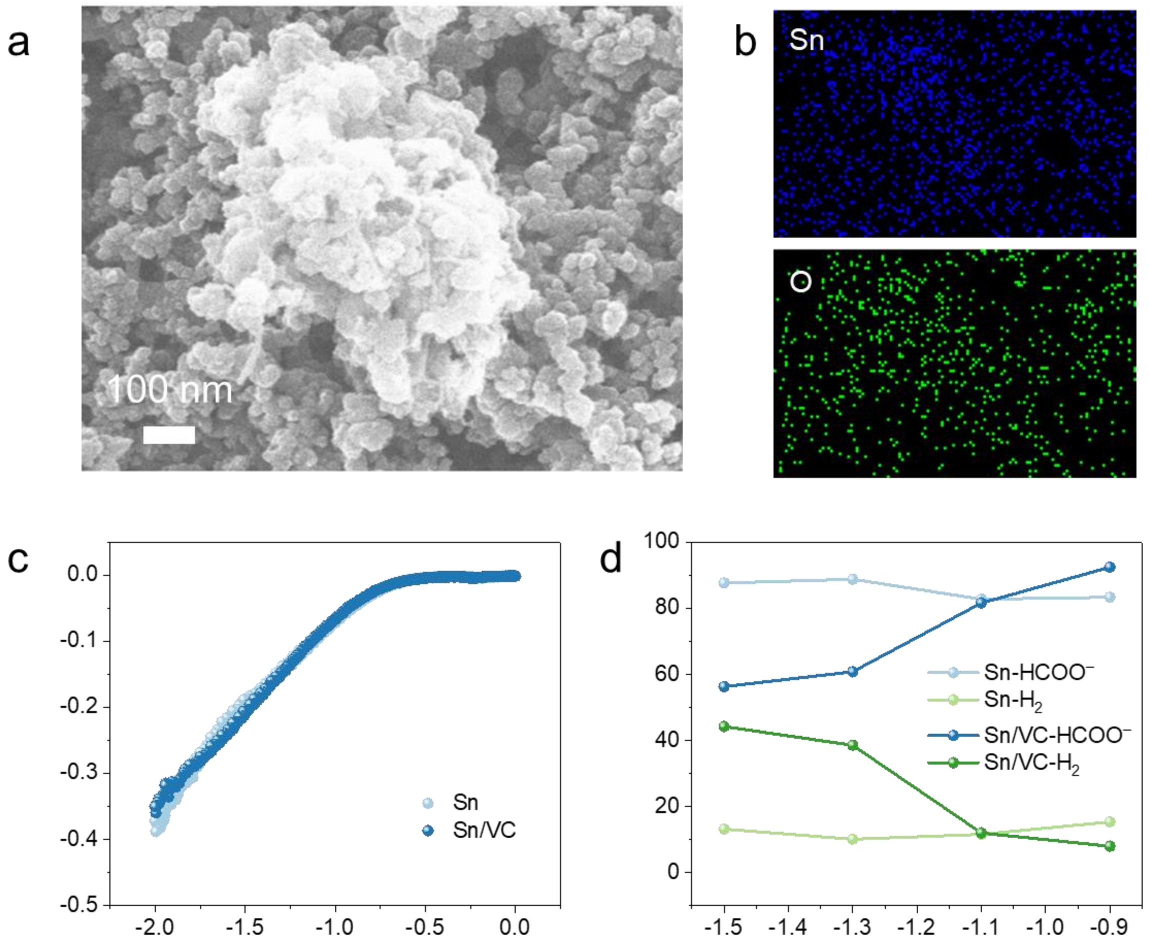

Figure S40. (a) SEM image of Sn/VC catalyst. Sn electrode was synthesized by sputtering metal Sn target on the gas diffusion electrode using a magnetron sputtering machine. The sputtering process was carried out at a power of 1 W for 10 min. Sn/VC was obtained by spraying VC dispersion solution on Sn electrode and dried in vacuum oven for 10 hours. (b) EDS mapping of Sn and O elements in Sn/VC electrode. (c) The LSV curves of Sn and Sn/VC electrode in alkaline flow-cell system with 1 M KOH. (d) The corresponding products of In and Sn/VC.

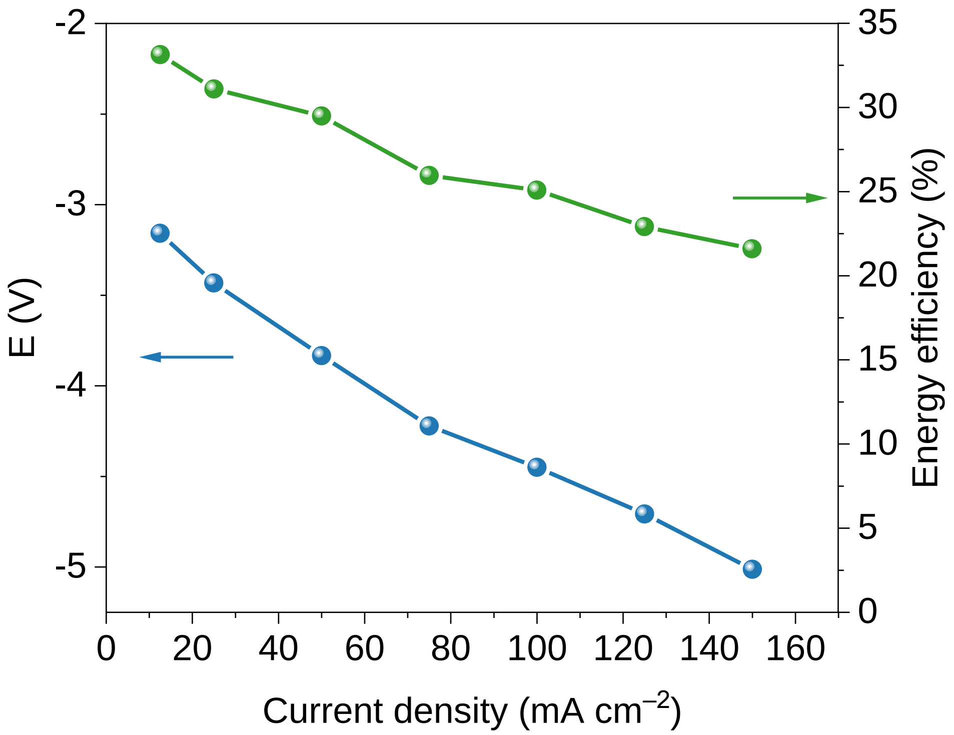

Figure S41. The total cell voltage and energy efficiency of formic acid as a function of current density.

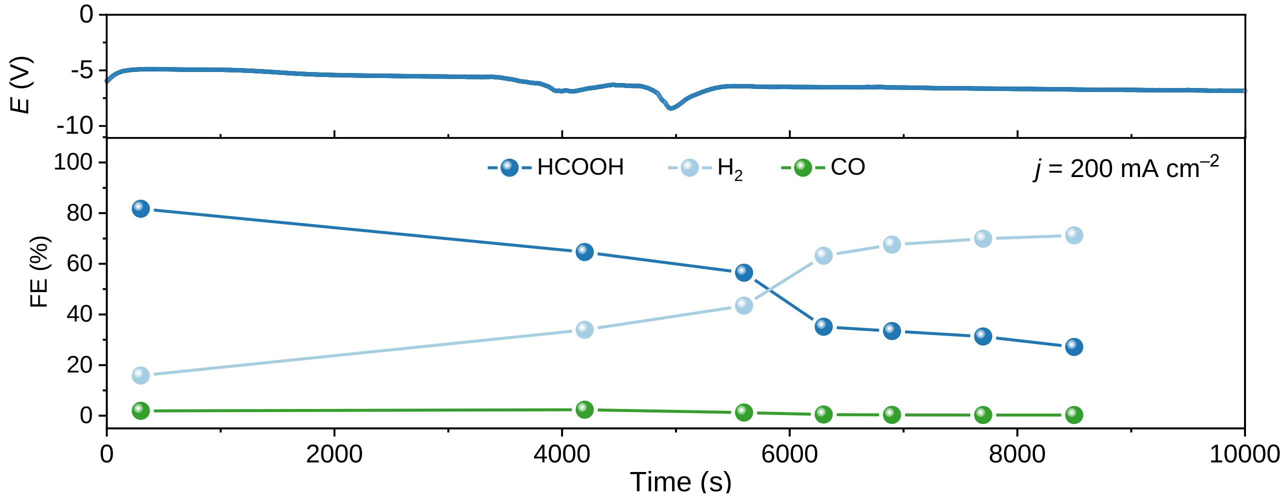

Figure S42. Long-term production of pure formic acid from the BS/VC at current densities of 200 mA cm^{-2}, in the all-solid-state reactor.

Table S1. Comparison of the potential range with Bi-based electrocatalysts recently reported – a high formate selectivity and the highest partial current density of formate for the BS/VC developed in this study.

Catalysts	Electrolytes	Potential range with formate selectivity over 90%	Highest partial current density of formate (mA cm^{-2})	FE (%)	Reference
BS/VC	1 M KOH	1.2 V	–910	94%	This work
2D-Bi[34]	2 M KOH	70 mV	–215	86%	Nature Energy, 2019, 4(9): 776-785
Defective Bi nanotubes[18]	1 M KOH	——	–206	98%	Nat. Commun., 2019, 10, 2807-2816
Bi NSs[33]	1 M KOH	200 mV	–360	89	Advanced Energy Materials, 2020, 10(36): 2001709
Bismuthene nanosheets[35]	1 M KHCO3	750 mV	–273	86%	Advanced Functional Materials, 2021, 31(4): 2006704
Bi_2O_3@C-800[36]	1 M KOH	700 mV	–208	92%	Angewandte Chemie, 2020, 132(27): 10899-10905
Bi_2S_3 nanoplatelets[37]	1 M KOH	80 mV	–211	100%	Journal of Materials Chemistry A, 2020, 8(25): 12385-12390
Bi RDs[22]	1 M KOH	360 mV	–289	94%	Advanced Materials, 2021, 33(31): 2008373
SnO_2-Bi_2O_3/C HNFs[38]	1 M KOH	——	–281	74%	Chemical Engineering Journal, 2021, 426: 131867
Bi-ene-NW[11]	1 M KOH	400 mV	–515	92%	Energy & Environmental Science, 2021, 14(9): 4998-5008
BOC@GDY[39]	1 M KOH	450 mV	–200	93.5%	Science Bulletin, 2021, 66(15): 1533-1541
Bi LNSs[12]	1 M KOH	660 mV	–165	89%	Small, 2021, 17(29): 2100602
Bi nanoribbons[40]	1 M KOH	550 mV	–193	95%	ACS Energy Letters, 2022, 7(4): 1454-1461
Bi nanobelts[41]	1 M KOH	900 mV	–331	96%	Advanced Functional Materials, 2022: 2201125

Defect Bi[42]	1 M KOH	400 mV	–151	85%	Chemical Communications, 2022, 58(22): 3621-3624

Supplementary References

1 Nørskov, J. K. *et al.* Origin of the overpotential for oxygen reduction at a fuel-cell cathode. *J. Phys. Chem. B* **108**, 17886-17892 (2004).

2 Njus, D., Kelley, P. M., Tu, Y.-J. & Schlegel, H. B. Ascorbic acid: The chemistry underlying its antioxidant properties. *Free Radical Biology and Medicine* **159**, 37-43 (2020).

3 Wu, Z. *et al.* Engineering bismuth–tin interface in bimetallic aerogel with a 3D porous structure for highly selective electrocatalytic CO_2 reduction to HCOOH. *Angew. Chem.* **133**, 12662-12667 (2021).

4 He, S. *et al.* The p-orbital delocalization of main-group metals to boost CO_2 electroreduction. *Angew. Chem.* **130**, 16346-16351 (2018).

5 Koh, J. H. *et al.* Facile CO_2 electro-reduction to formate via oxygen bidentate intermediate stabilized by high-index planes of Bi dendrite catalyst. *ACS Catal.* **7**, 5071-5077 (2017).

6 Chen, X. *et al.* Boron Dopant Induced Electron-Rich Bismuth for Electrochemical CO_2 Reduction with High Solar Energy Conversion Efficiency. *Small* **17**, 2101128 (2021).

7 Zhao, M. *et al.* Atom vacancies induced electron-rich surface of ultrathin Bi nanosheet for efficient electrochemical CO_2 reduction. *Appl. Catal. B-Environ.* **266**, 118625 (2020).

8 Yi, L. *et al.* Molten-salt-assisted synthesis of bismuth nanosheets for long-term continuous electrocatalytic conversion of CO_2 to formate. *Angew. Chem. Int. Edit.* **59**, 20112-20119 (2020).

9 Yang, F. *et al.* Bismuthene for highly efficient carbon dioxide electroreduction reaction. *Nat. Commun.* **11**, 1-8 (2020).

10 Cao, C. *et al.* Metal–organic layers leading to atomically thin bismuthene for efficient carbon dioxide electroreduction to liquid fuel. *Angew. Chem. Int. Edit.* **59**, 15014-15020 (2020).

11 Zhang, M. *et al.* Engineering a conductive network of atomically thin bismuthene with rich defects enables CO_2 reduction to formate with industry-compatible current densities and stability. *Energ. Environ. Sci.* **14**, 4998-5008 (2021).

12 Wang, D. *et al.* CO_2 Electroreduction to Formate at a Partial Current Density up to 590 mA mg^{-1} via Micrometer-Scale Lateral Structuring of Bismuth Nanosheets. *Small* **17**, 2100602 (2021).

13 Zhao, Y. *et al.* Spontaneously Sn-Doped Bi/BiO_x Core–Shell Nanowires Toward High-Performance CO_2 Electroreduction to Liquid Fuel. *Nano Lett.* **21**, 6907-6913 (2021).

14 Pang, R. *et al.* Tracking structural evolution: Operando regenerative CeOx/Bi interface structure for high-performance CO_2 electroreduction. *Natl. Sci. Rev.* **8**, nwaa187 (2021).

15 Yang, H. *et al.* Selective CO_2 reduction on 2D mesoporous Bi nanosheets. *Adv. Energy Mater.* **8**, 1801536 (2018).

16 Zhang, W. *et al.* Liquid-phase exfoliated ultrathin Bi nanosheets: uncovering the origins of enhanced electrocatalytic CO_2 reduction on two-dimensional metal nanostructure. *Nano*

Energy **53**, 808-816 (2018).
17 Lu, P. *et al.* Facile synthesis of a bismuth nanostructure with enhanced selectivity for electrochemical conversion of CO2 to formate. *Nanoscale* **11**, 7805-7812 (2019).
18 Gong, Q. *et al.* Structural defects on converted bismuth oxide nanotubes enable highly active electrocatalysis of carbon dioxide reduction. *Nat. Commun.* **10**, 1-10 (2019).
19 Fan, K. *et al.* Curved surface boosts electrochemical CO_2 reduction to formate via bismuth nanotubes in a wide potential window. *ACS Catal.* **10**, 358-364 (2019).
20 Wang, L. *et al.* Enhanced catalytic activity and stability of bismuth nanosheets decorated by 3-aminopropyltriethoxysilane for efficient electrochemical reduction of CO_2. *Appl. Catal. B-Environ.* **298**, 120602 (2021).
21 Peng, C.-J. *et al.* Hydrangea-like superstructured micro/nanoreactor of topotactically converted ultrathin bismuth nanosheets for highly active CO_2 electroreduction to formate. *ACS Appl. Mater. Inter.* **13**, 20589-20597 (2021).
22 Xie, H. *et al.* Facet engineering to regulate surface states of topological crystalline insulator bismuth rhombic dodecahedrons for highly energy efficient electrochemical CO_2 reduction. *Adv. Mater.* **33**, 2008373 (2021).
23 Wang, Y. *et al.* Rich Bismuth-Oxygen Bonds in Bismuth Derivatives from Bi_2S_3 Pre-Catalysts Promote the Electrochemical Reduction of CO_2. *Chem. Electro. Chem.* **7**, 2864-2868 (2020).
24 Wulan, B. *et al.* Electrochemically Driven Interfacial Transformation For High-Performing Solar-To-Fuel Electrocatalytic Conversion. *Adv. Energy Mater.*, 2103960 (2022).
25 Jia, L. *et al.* Phase-Dependent Electrocatalytic CO_2 Reduction on Pd3Bi Nanocrystals. *Angew. Chem.* **133**, 21909-21913 (2021).
26 Liu, S.-Q. *et al.* Electronic delocalization of bismuth oxide induced by sulfur doping for efficient CO_2 electroreduction to formate. *ACS Catal.* **11**, 7604-7612 (2021).
27 Fan, T. *et al.* Achieving high current density for electrocatalytic reduction of CO_2 to formate on bismuth-based catalysts. *Cell Reports Physical Science* **2**, 100353 (2021).
28 Yang, X. *et al.* Partial sulfuration-induced defect and interface tailoring on bismuth oxide for promoting electrocatalytic CO_2 reduction. *J. Mater. Chem. A* **8**, 2472-2480 (2020).
29 Ávila-Bolívar, B. *et al.* CO_2 reduction to formate on an affordable bismuth metal-organic framework based catalyst. *Journal of CO_2 Utilization* **59**, 101937 (2022).
30 Lv, W. *et al.* Electrodeposition of nano-sized bismuth on copper foil as electrocatalyst for reduction of CO_2 to formate. *Appl. Surf. Sci.* **393**, 191-196 (2017).
31 Yang, Z. *et al.* MOF derived bimetallic CuBi catalysts with ultra-wide potential window for high-efficient electrochemical reduction of CO_2 to formate. *Appl. Catal. B-Environ.* **298**, 120571 (2021).
32 Zhang, X. *et al.* Highly efficient and durable aqueous electrocatalytic reduction of CO_2 to HCOOH with a novel bismuth–MOF: experimental and DFT studies. *J. Mater. Chem. A* **8**, 9776-9787 (2020).
33 Yang, J. *et al.* Bi-based metal-organic framework derived leafy bismuth nanosheets for carbon dioxide electroreduction. *Adv. Energy Mater.* **10**, 2001709 (2020).
34 Xia, C. *et al.* Continuous production of pure liquid fuel solutions via electrocatalytic CO_2 reduction using solid-electrolyte devices. *Nat. Energy* **4**, 776-785 (2019).
35 Ma, W. *et al.* Monoclinic Scheelite Bismuth Vanadate derived Bismuthene nanosheets with rapid kinetics for electrochemically reducing carbon dioxide to formate. *Adv. Funct. Mater.*

31, 2006704 (2021).

36 Deng, P. *et al.* Metal–Organic Framework-Derived Carbon Nanorods Encapsulating Bismuth Oxides for Rapid and Selective CO_2 Electroreduction to Formate. *Angew. Chem.* **132**, 10899-10905 (2020).

37 Ding, P. *et al.* Simultaneous power generation and CO_2 valorization by aqueous Al–CO_2 batteries using nanostructured Bi_2S_3 as the cathode electrocatalyst. *J. Mater. Chem. A* **8**, 12385-12390 (2020).

38 Wang, X. *et al.* Carbon sustained SnO_2-Bi_2O_3 hollow nanofibers as Janus catalyst for high-efficiency CO_2 electroreduction. *Chem. Eng. J.* **426**, 131867 (2021).

39 Tang, S.-F. *et al.* Decorating graphdiyne on ultrathin bismuth subcarbonate nanosheets to promote CO_2 electroreduction to formate. *Sci. Bull.* **66**, 1533-1541 (2021).

40 Li, Y. *et al.* In Situ Confined Growth of Bismuth Nanoribbons with Active and Robust Edge Sites for Boosted CO_2 Electroreduction. *ACS Energy Lett.* **7**, 1454-1461 (2022).

41 Zeng, G. *et al.* Reconstruction of Ultrahigh-Aspect-Ratio Crystalline Bismuth–Organic Hybrid Nanobelts for Selective Electrocatalytic CO_2 Reduction to Formate. *Adv. Funct. Mater.*, 2201125 (2022).

42 Wang, Y. *et al.* Bismuth with abundant defects for electrocatalytic CO_2 reduction and Zn–CO_2 batteries. *Chem. Commun.* **58**, 3621-3624 (2022).
